# Diindeno[1,2-*b*:2′,1′-*n*]perylene: a closed shell related Chichibabin's hydrocarbon, the synthesis, molecular packing, electronic and charge transport properties†
†Electronic supplementary information (ESI) available. CCDC 1050080–1050082. For ESI and crystallographic data in CIF or other electronic format see DOI: 10.1039/c5sc00652j



**DOI:** 10.1039/c5sc00652j

**Published:** 2015-03-26

**Authors:** Kamal Sbargoud, Masashi Mamada, Jérôme Marrot, Shizuo Tokito, Abderrahim Yassar, Michel Frigoli

**Affiliations:** a UMR CNRS 8180 , UVSQ , Institut Lavoisier de Versailles , 45 Avenue des Etats-Unis , 78035 Versailles Cedex , France . Email: michel.frigoli@uvsq.fr; b Graduate School of Science and Engineering , Yamagata University , Yonezawa , Yamagata 992-8510 , Japan . Email: mamada@yz.yamagata-u.ac.jp; c UMR CNRS 7647 , LPICM-École Polytechnique , 91128 Palaiseau Cedex , France . Email: abderrahim.yassar@polytechnique.edu

## Abstract

Diindeno[1,2-*b*:2′,1′-*n*]perylene, a new derivative of the indenoacene family was synthesized, and its electronic, electrochemical, and electrical properties were investigated. This material has a closed shell electronic configuration which corresponds to a quinoidal structure with a low band gap of 1.35 eV. Molecular packing in the single crystal was studied by single-crystal X-ray structural analysis, and this information was subsequently used in the determination of the charge transfer integrals *via* density functional theory methods. The charge-carrier transport properties of the diindeno[1,2-*b*:2′,1′-*n*]perylene-5,12-dione and diindeno[1,2-*b*:2′,1′-*n*]perylene derivatives were investigated through the fabrication and characterization of field-effect transistors *via* both vacuum-deposited and solution-processed films, respectively. Diindeno[1,2-*b*:2′,1′-*n*]perylene exhibited a field-effect behaviour with a hole mobility up to 1.7 × 10^–3^ cm^2^ V^–1^ s^–1^ when the active layer was solution-processed.

## Abstract 2

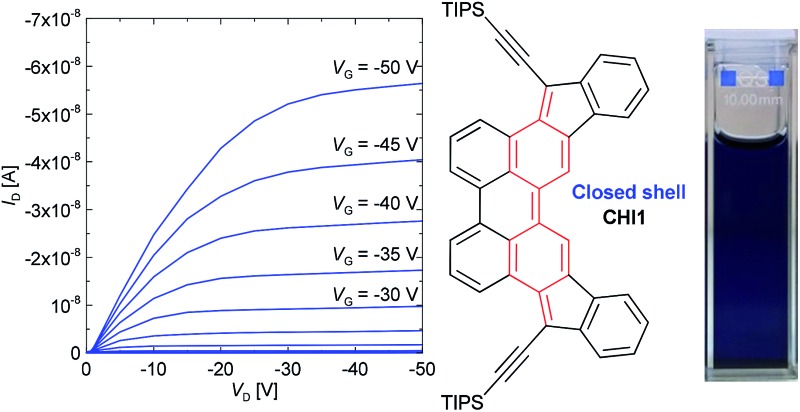
A fixed Chichibabin's hydrocarbon **CHI1** shows a closed shell configuration with a broad absorption from 400 up to 900 nm.

## Introduction

**Fig. 1:**
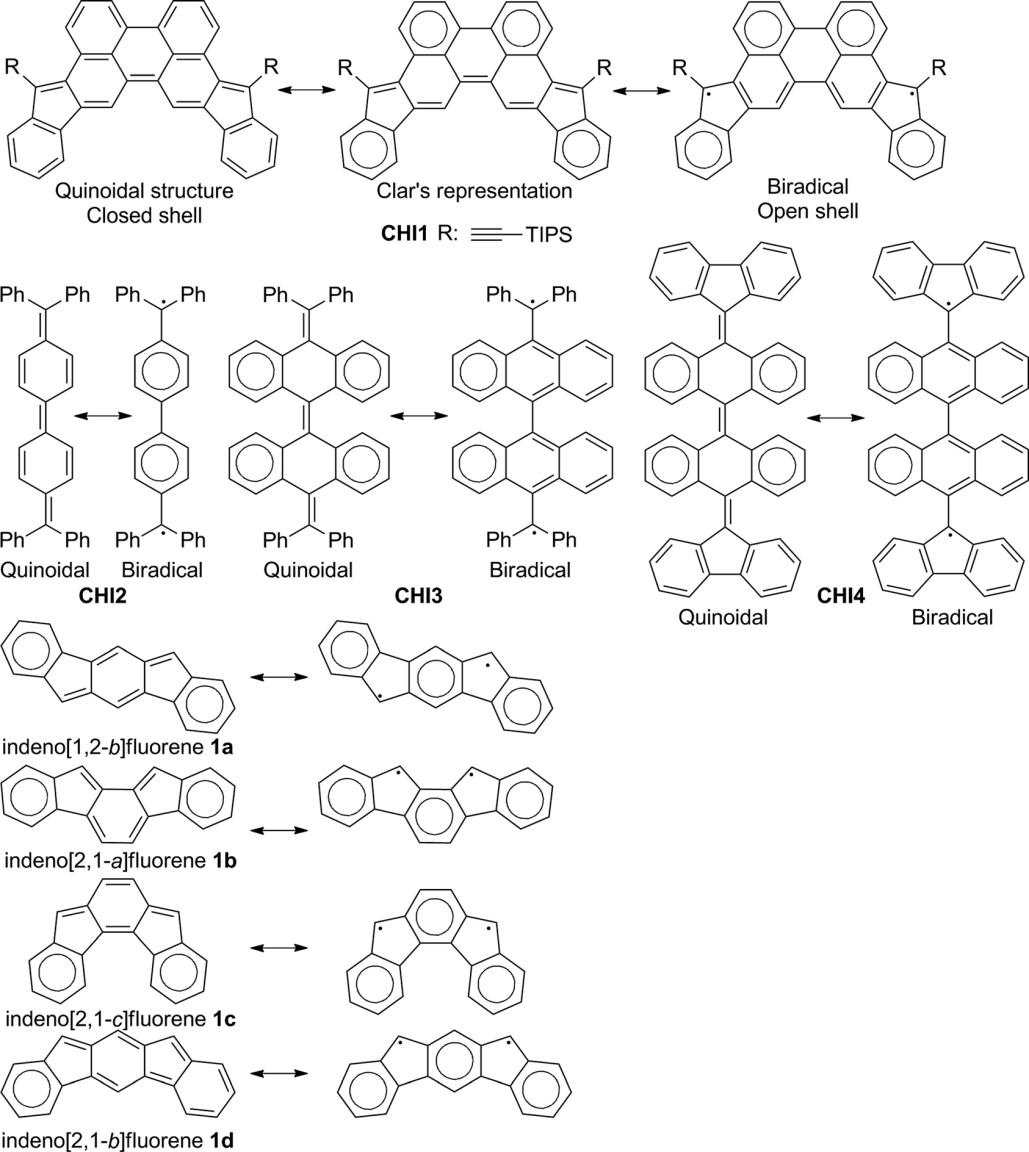
Chemical and resonance structures of the indenofluorene family, the Chichibabin's **CHI2**, the tetrabenzo-Chichibabin's derivatives **CHI3** and **CHI4** and the diindeno[1,2-*b*:2′,1′-*n*]perylene **CHI1** reported in this work.

The design and synthesis of new functional π-conjugated materials is a major issue for the development of the next generation of organic optoelectronic devices. Continuous research efforts have contributed to the great advances in the development of materials, along with innovations and optimizations of the device structure and processing.^1,2^ The progresses made are the result of the improvements in the fabrication processes and a better understanding of the design rules, yielding efficient π-conjugated materials. Tremendous efforts have been focused on the functionalization of known molecules (*e.g.* thiophene, benzene, fluorene, *etc.*) with electron-withdrawing or donating groups, alternating electron donors and electron deficient building blocks or the incorporation of heteroatoms into the π-conjugated backbone.^3^ However, further improvement of the material performance requires the development of a new concept in the designing of building blocks and an in-depth understanding of the structure–property relationships. Toward this end, several synthetic approaches have been developed, with a promising one being the use of quinoidal molecules as a useful building block for constructing functional materials. Quinoidal polycyclic hydrocarbons (QPHs) have recently been the subject of intense research due to their potential to have an open shell character with fascinating optical, electronic and magnetic properties.^4^ Environmentally stable closed shell QPHs are very promising for OFETs and photovoltaic applications due to their inherent planar structure that could improve the π–π stacking capability and facilitate charge delocalization and transport. Moreover, the quinoidal π-conjugated system reduces the HOMO–LUMO energy gap by stabilizing the LUMO energy level more than it destabilizes the HOMO level compared to the aromatic analogues and shows amphoteric redox behaviours, which is a prerequisite for their use as ambipolar semiconductors. Several QPHs have been developed in recent years, such as bisphenalenyls,^5^ zethrenes^6^ and indenofluorenes.^7^ The latter family is of interest due to the ease of their synthesis and the tuning of their optical and electronic properties, with the possibility to fuse two indene units in different fashions into a diverse π-conjugated spacer. Considering a benzene group as the conjugated spacer, four isomers with a plane or centre of symmetry have been developed (Fig. 1).^7a–d,f^ They have either a central *para*- (**1a**, **1c**), *ortho*- (**1b**) or *metha*-quinomethane (**1d**) unit. Isomers **1a–c** are best described as quinoidal closed shell molecules in the ground state, ensuring a good stability, whereas **1d** only has a little contribution of the singlet biradical canonical structure to the ground-state electronic configuration. The difference between these isomers is best described by considering the number of aromatic sextets between the quinoidal form (closed shell) and the biradical form (open shell) according to Clar's rule. The closed shell **1a–c** has two aromatic sextets whereas the corresponding open shell has three aromatic sextets. The gain in aromaticity in the corresponding open shell resonance is not enough to compensate the dissociation energy of the two double bonds and the reorganization of the π-electrons in the system. For **1d**, the biradical form has two more aromatic sextets compared to the quinoidal one and leads to a little contribution from the biradical resonance in the ground state. The biradical character of molecules belonging to the bisindeno-acene family can be simply anticipated when a difference of at least two aromatic benzenoid sextets between the quinoidal and biradical form is present.

Despite the progress made on synthesizing indenofluorene materials with good optical and electronic properties, only three reports describing the organic field effect transistor (OFET) characteristics have been reported. Low carrier mobilities (hole and electron) of 8.2 × 10^–6^ cm^2^ V^–1^ s^–1^ were measured for aryl-substituted derivatives in evaporated thin films.^7*b*^ The low mobilities were attributed to the lack of efficient π–π stacking in the crystal. A higher hole mobility (1.6 × 10^–2^ cm^2^ V^–1^ s^–1^) was obtained from a preliminary field effect transistor test on the spin-coated thin films of a diindenothienothiophene derivative.^7*h*^ Single crystal-OFETs with hole and electron mobilities up to 7 × 10^–4^ and 3 × 10^–3^ cm^2^ V^–1^ s^–1^, respectively, were reported too.^7*c*^ However, single crystal devices might be not well suited for practical applications in large area electronics due to their processing limitations.

In an effort to gain a deeper insight into the electronic structure of the indenoacene family and to further expand their versatility scaffold, we are interested in other conjugated systems that are larger than previously reported. Thus, from the viewpoint of molecular design, we introduce indeno groups into the perylene scaffold for several reasons: (a) the diindeno[1,2-*b*:2′,1′-*n*]perylene **CHI1** (Fig. 1) can be regarded as a fixed Chichibabin's hydrocarbon **CHI2**, which was recently found to exist in a semi-quinoid structure, with a significant contribution of the singlet biradical resonance form (Fig. 1).^8^ Moreover, two recent tetrabenzo-Chichibabin's derivatives have been disclosed, displaying either a closed (**CHI3**) or an open shell (**CHI4**) electronic configuration (Fig. 1).^9^ For these cases, even though the closed shell form has two more aromatic sextets than the open shell one, the electronic configuration could be tuned by a substituent effect on the thermodynamic stabilization of the biradical form. **CHI1** can be viewed also as an extension of the closed shell indeno[2,1-*c*]fluorene **1c** derivative in which the central phenylene group is replaced by a *p*-biphenylene linker. Therefore, the extension of the π-conjugation should lead to a low band gap material; (b) according to Clar's rule, the quinoidal structure of **CHI1** and the biradical resonance form have the same number of aromatic sextets (Fig. 1). So, in line with the relationship between the structure and the electronic configuration known for the indenofluorene family as described above, **CHI1** should have a closed shell configuration ensuring a good stability. Thus, it is of high interest to assess the electronic configuration of **CHI1** in order to a have better understanding of the relationship between the structure and the electronic configuration; (c) the perylene scaffold should promote good π–π overlap, thus good charge transport properties could be expected.

Herein, we describe an efficient synthetic method to prepare **CHI1**, in which the conjugation of the central perylene core is extended by the annelation of the two indene units. To address the processability issue, we introduce the (triisopropylsilyl)acetylene group to both improve the solvent solubility, stabilizing the structure by an electronic effect, increase the π–π interactions and improve the charge transport properties.^10^ An overview of the synthetic procedures is presented first and then the electronic and electrical properties of these new materials, including their absorption, electrochemical properties, molecular packing and charge transport behaviour, are developed and discussed.

## Results and discussions

**Scheme 1:**
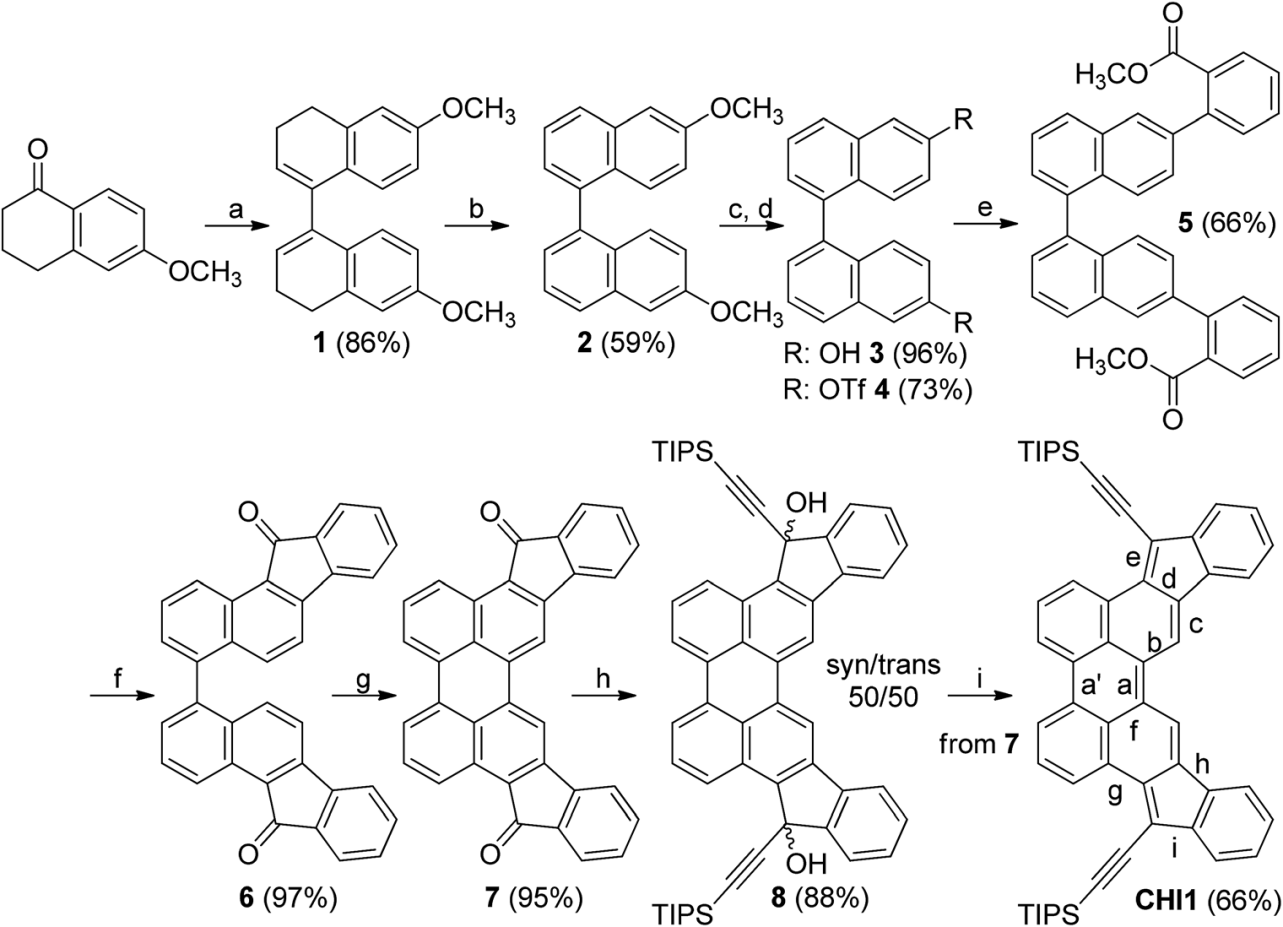
Synthetic path for **CHI1**: (a) Zn, TMSCl, HCl, THF –50 °C; (b) (1) trityl fluoroborate, CH_2_Cl_2_, 0 °C; (2) NEt_3_, rt; (c) BBr_3_, CH_2_Cl_2_, 0 °C; (d) OTf_2_, pyridine, CH_2_Cl_2_, 0 °C; (e) 2-methoxycarbonylphenylboronic acid, Pd(PPh_3_)_4_, K_3_PO_4_, DMF, 95 °C; (f) CH_3_SO_3_H, 75 °C; (g) K_2_CO_3_, ethanolamine, 160 °C; (h) TIPSCCLi, THF, 0 °C – rt; (i) SnCl_2_, toluene, 120 °C.

The synthetic path used to synthesize **CHI1** followed the same strategy as the synthesis of indenofluorene derivatives which relies on the synthesis of a diketone molecule that is consequently reacted with an appropriate lithium derivative, followed by a dehydroxylation reaction (Scheme 1). However, the preparation of **CHI1** was more challenging than that of the other indenoacene derivatives reported so far due to the need to construct the perylene core with suitable functionalities. One of the simplest and efficient methodologies to construct the perylene moiety is based on a base-promoted cyclodehydrogenation reaction of the 1,1′-binaphthyl derivatives bearing withdrawing substituents at the *peri*-position using K_2_CO_3_ and ethanolamine.^11,12^ We assumed that this procedure should work well with the ketone groups positioned in *para* of the reactive carbons in which the cyclisation occurs. Consequently, the critical reaction involved the preparation of the diindeno[1,2-*b*:2′,1′-*n*]perylene-5,12-dione **7** from the 1,1′-binaphthyl derivative **6**, namely 11*H*,11′*H*-[4,4′-bibenzo[*a*]fluorene]-11,11′-dione.

The synthesis starts with the dimerization of 6-methoxytetralone using zinc in presence of protic chlorotrimethylsilane.^13^ It should be noted that with a simple filtration under a silica gel pad, the 6,6′-dimethoxy-3,3′,4,4′-tetrahydro-1,1′-binaphthalene **1** decomposes partially to the corresponding hexahydrobenzo[*j*]fluoranthene derivative involving a ring closure with the formation of 5-membered ring. Nevertheless, compound **1** was isolated with a good quality and a yield of 86% after workup without purification. The aromatisation of compound **1** could not be carried out with a classical oxidant such DDQ and *p*-chloranil. Thus, the 6,6′-dimethoxy-1,1′-binaphthalene **2** was obtained in 59% yield using trityl fluoroborate instead. Treatment of **2** with BBr_3_ quantitatively yielded the corresponding dihydroxybinaphthalene **3**, followed by a triflation reaction carried out in a classical manner which afforded the corresponding 1,1′-binaphthalene triflate **4**. The palladium-catalysed Suzuki–Miyaura cross-coupling reaction between the bis-triflate **4** and 2-methoxycarbonylphenylboronic acid gave compound **5** in a 66% yield. The cyclization of the ester group was carried out with methanesulfonic acid to furnish the dione **6** in a quantitative yield. The cyclodehydrogenation of **6** was efficiently done with K_2_CO_3_/ethanolamine upon heating to give the desired parent diindenoperylene-5,12-dione **7**. The addition of the lithium-triisopropylsilylacetylene to **7** induced the formation of the diols **8** in excellent yield. The corresponding *syn*-/*anti*-diastereoisomers were easily separated under column chromatography as the difference in the retention factor (*R*_f_) is large. The *R*_f_ values were found to be 0.39 and 0.09 in a mixture of petroleum ether/dichloromethane for the first and second diastereoisomer respectively. The SnCl_2_-mediated dehydroxylation of the resulting mixture of diols was carried out in toluene at 120 °C for 1 h, leading to a dark blue solution. A longer time induces the formation of by-products. The target molecule **CHI1** was isolated in a 66% yield over two steps after purification under a silica gel column in a mixture of toluene/petroleum ether (50/50). Due to the very low solubility of compounds **6** and **7** in common solvents, only the ^1^H NMR spectrum of **7** could be recorded. However, these two molecules were characterized by infrared spectroscopy, X-ray crystallography and elemental analysis.^14,15^ Infrared spectra of compounds **7** and **8** showed the characteristic peaks of C=O stretching belonging to an arylketone at 1695 cm^–1^ and 1691 cm^–1^, respectively (see ESI†). Other signals related to the formation of a H-bonded carbonyl group structure are detected at 1601 cm^–1^ and 1579 cm^–1^ for **7** and 1605 cm^–1^ and 1579 cm^–1^ for **8**. The target molecule **CHI1** was fairly soluble in common organic solvents and was characterized by ^1^H NMR, infrared spectroscopy, elemental analysis and X-ray crystallography (see ESI†).^16^

**Fig. 2:**
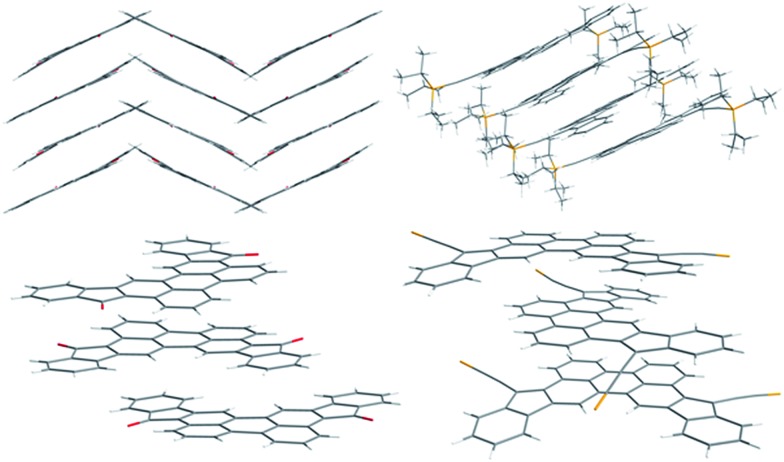
Crystal structures and molecular packing of **7** (left) and **CHI1** (right). The isopropyl groups were removed for clarity.

The ^1^H NMR spectrum of **CHI1** shows a sharp signal at room temperature and consequently supports the quinoidal structure of the ground state (see ESI†). Crystals suitable for X-ray structure analysis were obtained by recrystallization from chloroform for **7** and benzene for **CHI1** (Fig. 2). Both compounds gave crystals with an orthorhombic symmetry. **7** and **CHI1** crystallize in the *Pbcn* and *Pbca* space groups, respectively. It should be noted that **CHI1** co-crystallized with benzene molecules in a ratio 2 to 1. Selected bond lengths of the π-part, which changes significantly between **7** and **CHI1**, are listed in Table 1 together with those of Chichibabin's molecules **CHI2** and **CHI3**.

**Table 1:** Comparison of the bond lengths of **7** and **CHI1** in the crystals and calculated

Bond (Å)	**7**	**7** ^*a*^ (DFT)	**CHI1**	**CHI1** ^*a*^ ^,^ ^*b*^ (DFT)	**CHI2** ^*c*^	**CHI3** ^*d*^
*a*	1.467 (3)	1.474	1.406 (5)	1.414	1.448 (4)	1.350 (8)
*a*′	1.466 (4)	1.474	1.470 (5)	1.462		
*b*	1.389 (3)	1.400	1.435 (5)	1.439	1.420 (3)	1.496 (6)
*c*	1.388 (3)	1.394	1.352 (5)	1.359	1.372 (3)	1.401 (5)
*d*	1.378 (4)	1.393	1.450 (5)	1.452	1.429 (3)	1.482 (6)
*e*	1.484 (4)	1.497	1.386 (5)	1.393	1.420 (3)	1.342 (6)
*f*	1.435 (3)	1.434	1.451 (5)	1.447		
*g*	1.413 (4)	1.414	1.438 (5)	1.449		
*h*	1.484 (4)	1.485	1.467 (5)	1.464		
*i*	1.496 (4)	1.497	1.467 (5)	1.472		

^*a*^Calculated with the DFT method at the B3LYP/6-31G(d,p) level using the Gaussian 09 program.^17^

^*b*^The TIPS groups of **CHI1** were omitted and hydrogen atoms were used in their place.

^*c*^Taken from ref. 8b.

^*d*^Taken from ref. 9.

For comparison, the theoretical bond lengths of **7** and **CHI1** without the TIPS groups estimated by the DFT calculations are given in Table 1 as well. The calculated bond lengths for both molecules agree well with those of the experimental data within experimental errors. The crystal structure of **7** shows characteristics of the perylene scaffold. By contrast, the structure of **CHI1** shows clearly a bond-length alternation in the π-skeleton: the bonds denoted by *a*, *c*, and *e* have substantial double-bond character, whereas the bonds denoted by *b*, *d* and *f*–*i* have more of a single-bond character (Table 1; Scheme 1). The bonds lengths (*d*, *e*, *h*, *i*) of the five-membered rings are typical for the closed shell quinoidal indenofluorene derivatives. The shortening of bond length *a* (1.406 (5) Å) compared to bond lengths *a*′ (1.470 (5) Å) and *a* (1.448 (4) Å) in the corresponding Chichibabin's molecule **CHI2** is significant but longer than the one observed for **CHI3** (1.350 (8) Å). The bond length of *a* in **CHI1** is longer than a typical double bond, which is ascribed to steric hindrance between the two hydrogens located in the bay region of the perylene core. The distance between them is less than the sum of their van der Waals radii. The bond lengths suggest unambiguously that **CHI1** has a quinoidal electronic configuration in the ground state. It should be noted that **CHI1** is stable in a non-degassed toluene solution for more than six months in the dark and for 1 week in the laboratory environment. Moreover, the analysis of the nucleus independent chemical shift (NICS) values, NICS(1) (NICS(1)zz),^18^ for the five-membered and the quinoidal adjacent rings were –1.80 (5.25) and –1.66 (4.04), respectively, indicates a weak antiaromatic character. In comparison, the isomer **1c** shows a stronger antiaromatic character, with values for the five-membered ring of 4.18 (21.76) and 0.57 (9.61), for the quinoidal ring (see ESI†).

Compounds, **7** and **CHI1**, arrange into a 1-D columnar stack in which the molecules form pairwise slipped stacks. The distance between the average planes within the pair is 3.37 Å and 3.39 Å and 3.19 Å and 3.41 Å within adjacent pairs for **7** and **CHI1** respectively. The lateral slip of the π-conjugated system was found to be 1.56 Å and 2.04 Å in the pair and 5.14 Å and 2.00 Å between pairs for **7** and **CHI1** respectively. In **7**, the 1D-columns form layers of parallel columns and each alternate layer is twisted to each other. The dihedral angle between the average planes is 54.28°. The 1D-columns interact through hydrogen bonds (CH–O) of length 2.47 Å into the layers and 2.43 Å within the layers. For **CHI1**, the 1D-columns are separated from the adjacent columns by the TIPS groups and benzene molecules.

The charge-transfer processes are impacted by the geometry and molecular packing as well as by the intermolecular electronic coupling. An accurate understanding of all these molecular characteristics is an important prerequisite for the design and selection of appropriate molecules, as well as for optimizing the performance of the devices. Therefore, we have performed a study of the transfer integral in the dimers of **7** and **CHI1** (Fig. S2 in ESI†) using the Amsterdam Density Functional (ADF) program package. Compound **7** exhibits a large intra-dimer transfer integral, of the order of 142 meV and 4.3 meV for inter-dimer contacts. Although compound **CHI1** displays a slightly different crystalline structure, the calculated transfer integral (5.4 meV) is much lower than the transfer integral of **7**. Hence, this study of the transfer integral shows clearly that the intra-columnar direction is a favourable direction for charge transport.

**Fig. 3:**
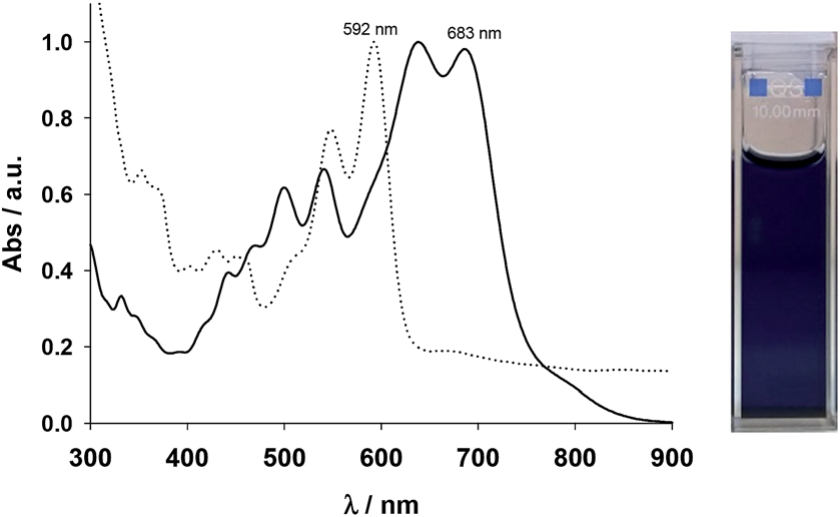
Absorption spectra of **7** (dotted line) and **CHI1** (bold line) in toluene. Insert is the photo of **CHI1**.

The UV-visible absorption spectra of **7** and **CHI1** are depicted in Fig. 3. Compound **7** was very insoluble in common solvents, even at a concentration of 2 × 10^–5^ mol L^–1^. At this concentration, the spectrum shows a maximum wavelength at 592 nm and a diffusion band indicating the presence of aggregates. Compound **CHI1** displays a strong blue colour in a toluene solution. The UV-vis spectrum of **CHI1** exhibits strong acene-like vibronic features in the visible energy range that extends into the near-IR region (900 nm), with a lower energy of *λ*_max_ at 683 nm.

**Fig. 4:**
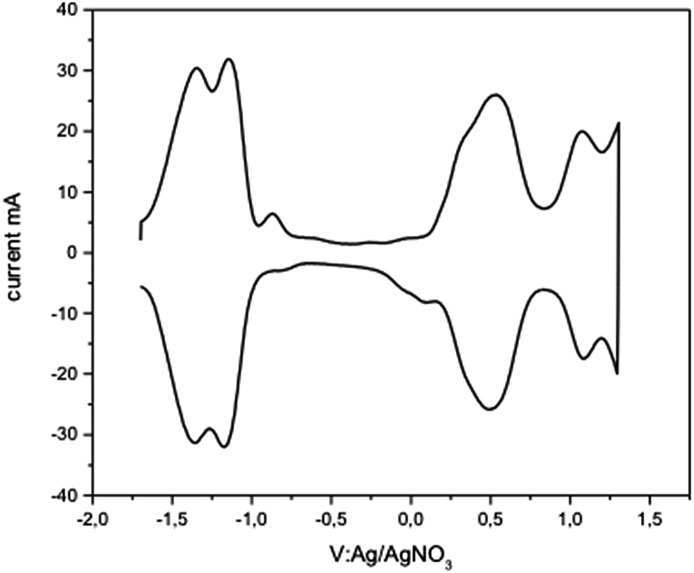
Square wave voltammogram of **CHI1** in a dichloromethane solution, scan rate 60 mV s^–1^, step potential 50 mV; square-wave frequency 1 Hz; and square-wave amplitude 10 mV.

Cyclic voltammetry and square wave voltammetry (SQW) were used to investigate the electrochemical behaviour and probe the HOMO/LUMO levels of **CHI1** (Fig. 4). The compound **CHI1** showed an amphoteric redox behaviour and exhibits two reversible oxidation and reduction peaks, which are typical for indenofluorene derivatives. In SQW, compound **CHI1** displayed two oxidation peaks at 0.5 and 1.1 V and two reduction peaks, *E*_red_, at –1.1 and –1.2 V (*vs.* Ag/AgNO_3_) using the Fc/Fc^+^ couple as the internal standard. The onset potentials of the first oxidation and reduction peaks were 0.35 and – 1 V respectively. The HOMO and LUMO levels were estimated from the onset potentials and converted to the vacuum scale according to the formula of HOMO = –(*E*_ox-onset_ + 4.8) eV, LUMO = –(*E*_red-onset_ + 4.8) eV.^19^ According to that, the HOMO and LUMO levels were found to be at –5.15 and –3.80 eV. Interestingly, **CHI1** and the TIPS-acetylene functionalised **1c** have a similar LUMO level (4.02 eV for **1c**). The extension of the conjugation of the central core and the number of aromatic rings in **CHI1** compared to **1c** (HOMO: 5.75 eV) leads to an increase of the HOMO level by 0.60 eV.

The charge transport properties of diindeno[1,2-*b*:2′,1′-*n*]perylene-5,12-dione (**7**) and diindenoperylene (**CHI1**) were investigated in bottom-gate/top-contact (BG/TC) and top-gate/bottom-contact (TG/BC) organic field-effect transistor geometries (details of the device fabrication are provided in the ESI,† Fig. S3).

**Fig. 5:**
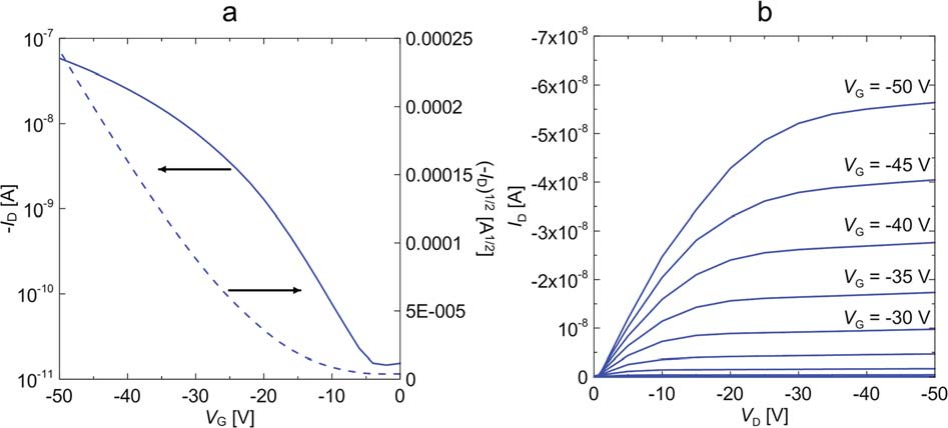
(a) Transfer and (b) output characteristics of the TG/BC device of **CHI1** deposited by drop-casting from a 0.2 wt% solution in mesitylene with 0.05 wt% of PS.

Both materials **7** (vacuum-deposited) and **CHI1** (solution-processed film) exhibited a measurable hole mobility and showed moderate charge-transport behaviour. Fig. S4 in the ESI† shows the transfer and output characteristics of an OFET. A hole mobility of 2 × 10^–5^ cm^2^ V^–1^ s^–1^ was extracted from *I*–*V* output characteristic for material **7**. When the substrate pre-set temperatures were increased, the device performances of **7** dramatically decreased, and no field effect was detected at *T*_sub_ = 90 °C. The low charge carrier mobility of **7** may be due to the unfavorable orientation of the crystallites and crystallite alignments with respect to the substrate, as evidenced by a preliminarily investigation of the crystallite orientation of the films using grazing incidence X-ray diffraction. Moreover, the poor performance of this compound might be also attributed to a discontinuous morphology, consisting of a spherical structure, possibly hindering the charge transport by numerous grain boundaries, as evidenced by atomic force microscopy (AFM) observations, (Fig. S5 in ESI†). The bottom gate and top contact (BG/TC) OFET device for the thin film of **CHI1** spin-coated from 0.3 wt% chloroform solution showed a hole mobility of 7.3 × 10^–4^ cm^2^ V^–1^ s^–1^ (Fig. S6 and S7 in ESI†). The OFET devices of **CHI1** were optimized and a slightly better mobility of 1.7 × 10^–3^ cm^2^ V^–1^ s^–1^ was obtained for the drop-casted thin film with the TG/BC configuration (Fig. 5).

**Fig. 6:**
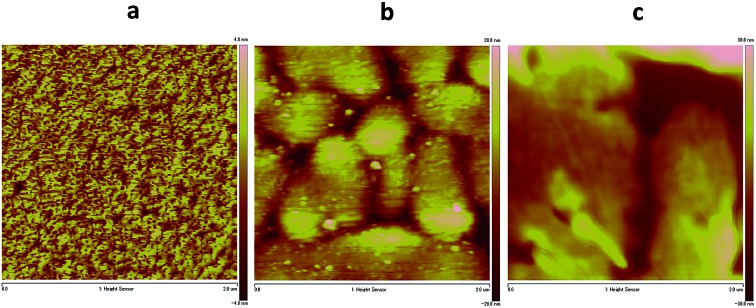
AFM images (2 µm × 2 µm) of thin films of **CHI1** deposited by (a) spin-coating from the chloroform solution on the HMDS-treated SiO_2_ substrate, (b) drop-casting from the mesitylene solution on the PVP substrate, and (c) drop-casting from the mesitylene solution with PS on the PVP substrate.

The *d*-spacing obtained from the first reflection peak of the out-of-plane X-ray diffraction (XRD) pattern for the **CHI1** thin film deposited on the HMDS-treated SiO_2_ substrate is 16.3 Å (2*θ* = 5.43°), which may be indexed as (102) based on the solved crystallographic structure of **CHI1**. The in-plane XRD of the **CHI1** thin film shows a reflection peak at 2*θ* = 26.0° (*d*-spacing of 3.42 Å) (Fig. S8 in ESI†), which corresponds to a *d*-spacing of π–π stacking distances between the indenoperylene cores in the crystal (3.39–3.41 Å in the bulk single crystal). The surface morphologies of the **CHI1** deposited on the HMDS-treated SiO_2_ and PVP substrates using different processing conditions were investigated using AFM. As shown in Fig. 6, the surface morphology of the film is strongly affected by the processing conditions, in particular, on the HMDS-treated SiO_2_ (Fig. 6a) the film shows a poor morphological order. On the other hand, on PVP substrates (Fig. 6b and c), the long-range order appears to be considerably improved and the films are characterized by the presence of larger circular islands. Consequently, the device performance under the different processing conditions is mainly affected by the changes in the surface morphology, as shown by the AFM measurements.

## Conclusions

In summary, we have developed an efficient method to prepare a new extended indenoacene derivative in which the two indene groups are introduced into a central perylene core. From a synthetic point of view, the base-promoted cyclodehydrogenation reaction of a 1,1′-binaphthyl derivative, bearing ketone groups positioned *para* to the reactive carbons in which the cyclisation occurs, using K_2_CO_3_ and ethanolamine is an efficient and versatile method to build up the functionalized perylene core. The novel diindeno[1,2-*b*:2′,1′-*n*]perylene **CHI1** has a closed shell electronic configuration in the ground state which confirms that the closed or open shell character of indenoacene derivatives can be predicted just by looking at the difference in the number of aromatic sextets between the two mesomeric forms. The diindeno[1,2-*b*:2′,1′-*n*]perylene has a quinoidal structure with a broad absorption from 400 nm up to 900 nm, a band gap of 1.35 eV and is packed into a 1-D columnar stack in the crystal. The best OFET performance, with mobility up to 1.7 × 10^–3^ cm^2^ V^–1^ s^–1^, has been found in devices based on solution processed thin films with a TG/BC configuration.
