# Supplementary material for: Diindeno[1,2-b:2′,1′-n]perylene: a closed shell related Chichibabin's hydrocarbon, the synthesis, molecular packing, electronic and charge transport properties
Source: Chem Sci. 2015 Mar 26;6(6):3402–9. doi: 10.1039/c5sc00652j (PMC5659070; doi:10.1039/c5sc00652j)
Supplement: Supplementary file 1 [file SC-006-C5SC00652J-s001.pdf]

Electronic Supplementary Material (ESI) for Chemical Science.
This journal is © The Royal Society of Chemistry 2015

Electronic Supplementary Information for:

Diindeno[1,2-*b*:2',1'-*n*]Perylene: a Closed Shell related Chichibabin's Hydrocarbon, Synthesis, Molecular Packing, Electronic and Charge Transport Properties

Kamal Sbargoud,[a] Masashi Mamada,[*b] Jérôme Marrot,[a] Shizuo Tokito[b], Abderahim Yassar[*c] and Michel Frigoli[*a]

[a] UMR CNRS 8180, UVSQ, Institut Lavoisier de Versailles, 45 avenue des Etats-Unis, 78035 Versailles Cedex, France

[b] Graduate School of Science and Engineering, Yamagata University, Yonezawa, Yamagata, 992-8510 Japan

[c] UMR CNRS 7647, LPICM-École Polytechnique, 91128 Palaiseau Cedex, France

General Methods for Synthesis **2**

Computational detail **2**

Instruments **10**

Synthesis **11**

X-ray structures **17**

11H,11'H-[4,4'-dibenzo[a]fluorene]-11,11'-dione 6 17

Diindeno[1,2-b:2',1'-n]perylene-5,12-dione 7 18

5,12-bis((triisopropylsilyl)ethynyl)diindeno[1,2-b:2',1'-n]perylene CHI1 19

NMR Spectra **20**

IR Spectra **28**

OFET Fabrication and Characterization **30**

General Methods for Synthesis

All reactions were carried out under argon. DCM was distilled from P_2O_5, THF and toluene were distilled over Na/benzophenone. DCM and toluene were kept over 3Å molecular sieves. All commercial reagents were used without further purification. ^{1}H and ^{13}C NMR spectra were recorded at room temperature on Brucker Avance-300 MHz NMR spectrometer. ^{1}H NMR spectra were recorded at 300 MHz and ^{13}C NMR spectra were recorded at 75 MHz. Chloroform residual peak was taken as internal reference at 7.27 ppm for ^{1}H NMR and 77 ppm for ^{13}C NMR. High-resolution mass spectra were obtained by using Waters Xevo Q-Tof using positive mode. Infrared spectra were recorded from Nicolet 6700 FT-IR spectrometer. The elemental analyses were carried out in the analysis laboratory of the elemental analysis department at CNRS-. Vernaison, France.

Computational detail

MO Calculations were performed using Gaussian 09 program.[1] The geometries were optimized at the B3LYP/6-31G(d,p) level (no imaginary frequencies). The triisopropylsilyl groups of **CHI1** were omitted and hydrogen atoms were used in their place. The B3LYP/6-31G(d,p) optimized structures were used for determining energies of the states for all calculations.

The time-dependent density functional theory (TD-DFT) calculations were conducted at the B3LYP/6-31G(d,p) level of theory. NICS values were estimated using the GIAO-B3LYP/6-31G(d,p) and GIAO-B3LYP/6-311+G(2df,2pd) methods. The open shell (OS) singlet were computed using broken symmetry UHF/6-31G(d,p), BS-UB3LYP/6-311+G(2df,2pd) and CASSCF(2,2) method. Triplet energies were calculated using UB3LYP/6-311+G(2df,2pd).

The transfer integrals were calculated using the PW-91/TZP level in the Amsterdam Density Functional (ADF) program.[2]

---

[1] Gaussian 09, Revision C.01: Frisch, M. J.; Trucks, G. W.; Schlegel, H. B.; Scuseria, G. E.; Robb, M. A.; Cheeseman, J. R.; Scalmani, G.; Barone, V.; Mennucci, B.; Petersson, G. A.; Nakatsuji, H.; Caricato, M.; Li, X.; Hratchian, H. P.; Izmaylov, A. F.; Bloino, J.; Zheng, G.; Sonnenberg, J. L.; Hada, M.; Ehara, M.; Toyota, K.; Fukuda, R.; Hasegawa, J.; Ishida, M.; Nakajima, T.; Honda, Y.; Kitao, O.; Nakai, H.; Vreven, T.; Montgomery, Jr., J. A.; Peralta, J. E.; Ogliaro, F.; Bearpark, M.; Heyd, J. J.; Brothers, E.; Kudin, K. N.; Staroverov, V. N.; Kobayashi, R.; Normand, J.; Raghavachari, K.; Rendell, A.; Burant, J. C.; Iyengar, S. S.; Tomasi, J.; Cossi, M.; Rega, N.; Millam, N. J.; Klene, M.; Knox, J. E.; Cross, J. B.; Bakken, V.; Adamo, C.; Jaramillo, J.; Gomperts, R.; Stratmann, R. E.; Yazyev, O.; Austin, A. J.; Cammi, R.; Pomelli, C.; Ochterski, J. W.; Martin, R. L.; Morokuma, K.; Zakrzewski, V. G.; Voth, G. A.; Salvador, P.; Dannenberg, J. J.; Dapprich, S.; Daniels, A. D.; Farkas, Ö.; Foresman, J. B.; Ortiz, J. V.; Cioslowski, J.; Fox, D. J. Gaussian, Inc., Wallingford CT, 2009.

[2] Amsterdam Density Functional (ADF) Code, Release 2012, Vrije Universiteit, Amsterdam, The Netherlands, 2012.

	**7**	**CHI1**
	Total Energy = −1455.85044562 a.u.	Total Energy = −1458.88516770 a.u.
**LUMO+1**	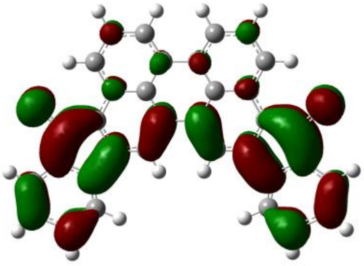 −2.547 eV	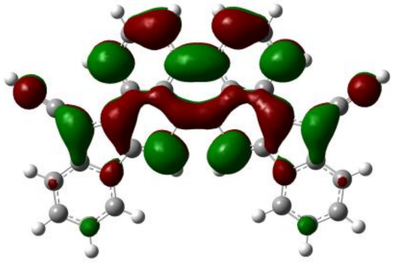 −2.214 eV
**LUMO**	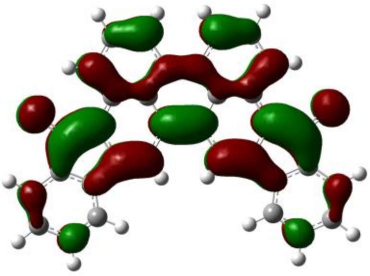 −3.405 eV	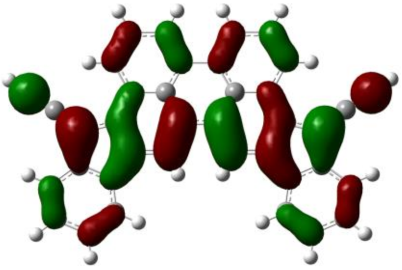 −3.458 eV
**HOMO**	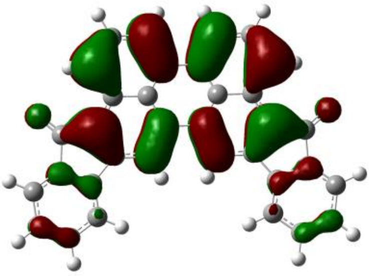 −5.611 eV	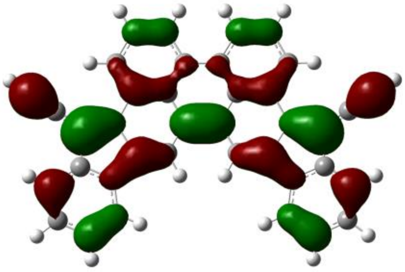 −5.249 eV
**HOMO−1**	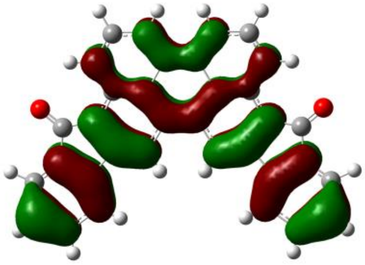 −6.639 eV	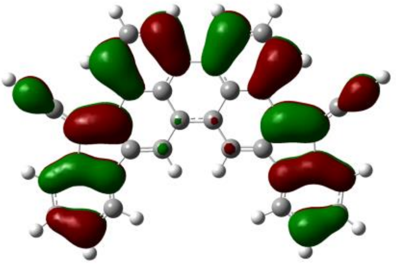 −5.744 eV

Figure S1. Molecular orbitals of **7** and **CHI1** calculated by DFT methods at the B3LYP/6-31+G(d,p)//B3LYP/6-31G(d,p) level of theory.

Table S1. Optimized geometry of **7** (B3LYP/6-31G(d,p))

Atom	X	Y	Z	Atom	X	Y	Z
O	-5.9626628	0.8661541	-0.0002286	O	5.9626626	0.8661542	0.0002279
C	-5.0258479	0.0818198	-0.0001106	C	5.0258478	0.0818199	0.0001099
C	-5.1191014	-1.4157611	0.0000339	C	5.1191014	-1.4157610	-0.0000344
C	-6.2423709	-2.2259412	0.0000681	C	6.2423709	-2.2259410	-0.0000682
H	-7.2344708	-1.7847525	-0.0000164	H	7.2344708	-1.7847524	0.0000161
C	-6.0553678	-3.6162211	0.0002147	C	6.0553679	-3.6162211	-0.0002145
H	-6.9151867	-4.2790346	0.0002458	H	6.9151869	-4.2790344	-0.0002453
C	-4.7669990	-4.1544197	0.0003216	C	4.7669991	-4.1544197	-0.0003212
H	-4.6385345	-5.2329102	0.0004352	H	4.6385347	-5.2329102	-0.0004343
C	-3.6316248	-3.3271251	0.0002850	C	3.6316248	-3.3271251	-0.0002849
H	-2.6379380	-3.7657641	0.0003701	H	2.6379381	-3.7657642	-0.0003696
C	-3.8181609	-1.9515227	0.0001399	C	3.8181609	-1.9515228	-0.0001402
C	-2.8563579	-0.8206854	0.0000650	C	2.8563578	-0.8206854	-0.0000650
C	-1.4625648	-0.8339178	0.0001087	C	1.4625648	-0.8339178	-0.0001086
H	-0.9508781	-1.7879959	0.0002319	H	0.9508781	-1.7879959	-0.0002318
C	-0.7370370	0.3642631	0.0000108	C	0.7370370	0.3642630	-0.0000108
C	-1.4428067	1.6132980	-0.0000717	C	1.4428068	1.6132980	0.0000720
C	-0.7372217	2.8596781	-0.0000816	C	0.7372218	2.8596780	0.0000819
C	-1.4755587	4.0394432	-0.0002394	C	1.4755588	4.0394431	0.0002397
H	-0.9700348	4.9974332	-0.0002948	H	0.9700350	4.9974332	0.0002951
C	-2.8810049	4.0394830	-0.0003411	C	2.8810050	4.0394828	0.0003414
H	-3.4101818	4.9877346	-0.0004609	H	3.4101819	4.9877344	0.0004613
C	-3.5849183	2.8571933	-0.0002822	C	3.5849183	2.8571930	0.0002823
H	-4.6678995	2.8358121	-0.0003421	H	4.6678994	2.8358118	0.0003422
C	-2.8844269	1.6247152	-0.0001461	C	2.8844269	1.6247151	0.0001462
C	-3.5598671	0.3824081	-0.0000772	C	3.5598671	0.3824081	0.0000771

Table S2. Optimized geometry of **CHI1** (B3LYP/6-31G(d,p))

Atom	X	Y	Z	Atom	X	Y	Z
C	-1.4396444	-1.5548932	-0.0000065	C	-4.7115429	4.2572942	0.0000261
C	-2.8776072	-1.5641807	-0.0000136	C	-3.5793472	3.4333202	0.0000141
C	-3.5834644	-0.2992416	-0.0000107	C	3.5793471	3.4333198	-0.0000256
C	-2.8149357	0.9327996	-0.0000034	C	4.7115427	4.2572940	-0.0000346
C	-1.4551350	0.9227191	0.0000038	C	5.9968247	3.7038553	-0.0000248
C	-0.7074427	-0.3068741	-0.0000001	C	6.1828724	2.3170996	-0.0000056
C	-0.7311926	-2.7909139	-0.0000052	C	-6.0689747	-0.8130269	-0.0000093
C	-1.4661045	-3.9923758	-0.0000104	C	6.0689759	-0.8130271	0.0000257
C	-2.8518759	-3.9896095	-0.0000205	C	7.0851448	-1.4777954	0.0000344
C	-3.5555136	-2.7879990	-0.0000232	C	-7.0851422	-1.4777973	-0.0000013
C	0.7074424	-0.3068742	-0.0000001	H	-7.9673673	-2.0750596	0.0000028
C	1.4396440	-1.5548936	0.0000034	H	7.9673702	-2.0750576	0.0000429
C	0.7311918	-2.7909140	0.0000014	H	-0.9325176	1.8704575	0.0000153
C	2.8776068	-1.5641814	0.0000076	H	-0.9538265	-4.9457925	-0.0000094
C	3.5555129	-2.7879999	0.0000117	H	-3.3925592	-4.9312743	-0.0000245
C	2.8518749	-3.9896103	0.0000101	H	-4.6380555	-2.7950388	-0.0000333
C	1.4661035	-3.9923762	0.0000047	H	4.6380549	-2.7950400	0.0000149
C	1.4551349	0.9227188	-0.0000037	H	3.3925580	-4.9312752	0.0000126
C	2.8149357	0.9327990	-0.0000033	H	0.9538253	-4.9457928	0.0000044
C	3.5834644	-0.2992423	0.0000068	H	0.9325178	1.8704572	-0.0000091
C	-4.9378605	0.0298769	-0.0000079	H	-7.1797690	1.8868604	0.0000131
C	-5.0578702	1.4966265	0.0000012	H	-6.8622044	4.3601097	0.0000378
C	-3.7562113	2.0535982	0.0000029	H	-4.5905456	5.3363048	0.0000359
C	3.7562114	2.0535977	-0.0000051	H	-2.5849848	3.8717842	0.0000121
C	5.0578705	1.4966261	0.0000053	H	2.5849846	3.8717836	-0.0000334
C	4.9378613	0.0298763	0.0000145	H	4.5905452	5.3363045	-0.0000508
C	-6.1828722	2.3170996	0.0000144	H	6.8622042	4.3601099	-0.0000342
C	-5.9968248	3.7038553	0.0000268	H	7.1797694	1.8868606	0.0000024

Table S3. Optimized geometry of **CHI1’** (diindeno[1,2-*b*:2',1'-*n*]perylene) (B3LYP/6-31G(d,p))

Atom	X	Y	Z	Atom	X	Y	Z
C	1.4567203	-0.8064405	0.0000040	C	3.6246686	-3.3011979	0.0000079
C	2.8135048	-0.8149850	0.0000058	C	4.7717501	-4.1067598	0.0000029
C	3.5794695	0.4258226	0.0000015	C	6.0464556	-3.5310931	-0.0000069
C	2.8703503	1.6906967	0.0000036	C	6.2087748	-2.1407578	-0.0000115
C	1.4359257	1.6815825	0.0000019	C	-6.2087751	-2.1407577	-0.0000020
C	0.7048082	0.4283262	0.0000021	C	-6.0464559	-3.5310930	0.0000002
C	3.5523093	2.9093847	0.0000074	C	-4.7717504	-4.1067597	0.0000024
C	2.8553562	4.1149799	0.0000063	C	-3.6246689	-3.3011979	0.0000025
C	1.4688633	4.1192629	0.0000023	H	0.9309059	-1.7525749	0.0000033
C	0.7313217	2.9191624	0.0000007	H	4.6375840	2.9127892	0.0000118
C	-0.7313214	2.9191626	-0.0000013	H	3.3978803	5.0555811	0.0000089
C	-1.4359255	1.6815826	-0.0000006	H	0.9579160	5.0734859	0.0000007
C	-0.7048080	0.4283263	0.0000011	H	-0.9309061	-1.7525749	0.0000004
C	-2.8703502	1.6906969	-0.0000013	H	-0.9579155	5.0734860	-0.0000060
C	-3.5794696	0.4258229	-0.0000007	H	-3.3978798	5.0555815	-0.0000061
C	-2.8135049	-0.8149849	0.0000008	H	-4.6375839	2.9127896	-0.0000041
C	-1.4567203	-0.8064404	0.0000009	H	5.7453578	0.8160632	-0.0000150
C	-1.4688629	4.1192631	-0.0000038	H	-5.7453580	0.8160635	-0.0000052
C	-2.8553558	4.1149802	-0.0000047	H	2.6383177	-3.7578100	0.0000158
C	-3.5523091	2.9093851	-0.0000032	H	4.6693821	-5.1877092	0.0000072
C	3.7761212	-1.9202284	0.0000025	H	6.9230572	-4.1724495	-0.0000108
C	5.0724962	-1.3314809	-0.0000064	H	7.2034334	-1.7035367	-0.0000189
C	4.9179046	0.1164176	-0.0000073	H	-7.2034337	-1.7035364	-0.0000042
C	-4.9179047	0.1164179	-0.0000028	H	-6.9230575	-4.1724494	0.0000005
C	-5.0724964	-1.3314808	-0.0000021	H	-4.6693824	-5.1877090	0.0000043
C	-3.7761212	-1.9202283	0.0000001	H	-2.6383179	-3.7578100	0.0000040

Table S4. Optimized geometry of **1a** (B3LYP/6-31G(d,p))

Atom	X	Y	Z	Atom	X	Y	Z
C	-4.6383637	-1.2108917	-0.0000028	C	4.6383637	1.2108916	-0.0000029
C	-5.5523662	-0.1517833	-0.0000046	C	5.5523660	0.1517831	-0.0000046
C	-5.1096082	1.1751818	-0.0000045	C	5.1096079	-1.1751819	-0.0000044
C	-3.7403101	1.4761945	-0.0000018	C	3.7403098	-1.4761946	-0.0000017
C	-2.8236933	0.4321125	0.0000012	H	-4.9876775	-2.2397541	-0.0000015
C	-3.2731995	-0.9212146	0.0000007	H	-6.6179165	-0.3614939	-0.0000059
C	-1.3568954	0.4006563	0.0000018	H	-5.8354397	1.9829493	-0.0000067
C	-0.9675064	-1.0167322	0.0000024	H	-3.4098015	2.5113871	-0.0000028
C	-2.1068115	-1.7886430	0.0000081	H	-2.1410570	-2.8724957	0.0000110
C	-0.4199141	1.3888934	0.0000005	H	-0.6965756	2.4404540	0.0000004
C	0.9675066	1.0167325	0.0000025	H	0.6965758	-2.4404540	0.0000002
C	1.3568955	-0.4006560	0.0000017	H	2.1410577	2.8724956	0.0000112
C	0.4199143	-1.3888933	0.0000003	H	4.9876776	2.2397539	-0.0000017
C	2.1068117	1.7886430	0.0000081	H	6.6179164	0.3614936	-0.0000060
C	3.2731996	0.9212145	0.0000006	H	5.8354393	-1.9829495	-0.0000067
C	2.8236932	-0.4321125	0.0000013	H	3.4098013	-2.5113872	-0.0000026

Table S5. Optimized geometry of **1c** (B3LYP/6-31G(d,p))

Atom	X	Y	Z	Atom	X	Y	Z
C	1.3907345	1.8832593	-0.0773550	C	4.1908789	-0.4793410	-0.1044137
C	0.6769587	3.1315872	-0.0505038	C	1.7347116	-1.8275486	0.2335412
C	-0.6769585	3.1315889	0.0503970	C	2.9491257	-2.5336042	0.2382868
C	-1.3907343	1.8832620	0.0772905	C	4.1617791	-1.8707849	0.0568466
C	-0.6839261	0.5838055	0.0006534	H	-3.5056261	2.4143930	0.2587459
C	0.6839262	0.5838054	-0.0006740	H	1.2389154	4.0598806	-0.0941734
C	-2.7373098	1.6545934	0.1698715	H	-1.2389151	4.0598839	0.0940349
C	-2.9903194	0.2247436	0.1022143	H	-5.1369686	0.0432455	0.2147055
C	-4.1908789	-0.4793373	0.1044302	H	-5.0909068	-2.4330695	-0.0579768
C	-4.1617792	-1.8707868	-0.0567823	H	-2.9379751	-3.6083494	-0.3919552
C	-2.9491258	-2.5336123	-0.2381999	H	-0.8153527	-2.3640721	-0.4245978
C	-1.7347117	-1.8275566	-0.2334786	H	3.5056261	2.4143840	-0.2588287
C	-1.7414244	-0.4520407	-0.0331054	H	5.1369685	0.0432381	-0.2147068
C	2.7373098	1.6545875	-0.1699283	H	0.8153526	-2.3640576	0.4246786
C	2.9903194	0.2247401	-0.1022221	H	2.9379750	-3.6083360	0.3920789
C	1.7414244	-0.4520396	0.0331207	H	5.0909067	-2.4330675	0.0580605

Table S6. Results on TD-DFT calculations of **7** at the B3LYP/6-31G(d,p) level

Excited State	Transition Energy	Main CI Coefficient	Oscillator Strength
1	1.9926 eV, 622.22 nm	0.6975 (HOMO → LUMO)	f = 0.2838
2	2.5916 eV, 478.40 nm	0.1872 (HOMO−1 → LUMO)	f = 0.0001
		0.6716 (HOMO → LUMO+1)	
3	2.7575 eV, 449.63 nm	0.6673 (HOMO−1 → LUMO)	f = 0.0129
		−0.1962 (HOMO → LUMO+1)	
4	2.8214 eV, 439.45 nm	0.6835 (HOMO−2 → LUMO)	f = 0.0379
		−0.1002 (HOMO → LUMO+3)	
5	2.8365 eV, 437.10 nm	0.2881 (HOMO−4 → LUMO+1)	f = 0.0000
		0.6350 (HOMO−3 → LUMO)	

Table S7 Results on TD-DFT calculations of **CHI1** at the B3LYP/6-31G(d,p) level

Excited State	Transition Energy	Main CI Coefficient	Oscillator Strength
1	1.8144 eV, 683.35 nm	0.2467 (HOMO−2 → LUMO)	f = 0.6506
		0.6696 (HOMO → LUMO)	
2	1.8502 eV, 670.10 nm	−0.1082 (HOMO−3 → LUMO)	f = 0.0186
		0.6897 (HOMO−1 → LUMO)	
3	2.4006 eV, 516.46 nm	0.6585 (HOMO−2 → LUMO)	f = 0.5228
		−0.2508 (HOMO → LUMO)	
4	2.4943 eV, 497.08 nm	0.5967 (HOMO−3 → LUMO)	f = 0.0027
		−0.3701 (HOMO → LUMO+1)	
5	2.8271 eV, 438.55 nm	0.5967 (HOMO−3 → LUMO)	f = 0.1412
		0.1284 (HOMO−1 → LUMO)	
		0.5747 (HOMO → LUMO+1)	

Table S8 NICS(0) and NICS(1) values of **1c**, **7** and **CHI1** calculated at the GIAO-B3LYP/6-31G(d,p) level and GIAO/6-311+G(2df,2pd)

Ring[a)]	**1c** (B3LYP/6-31G(d,p))				**1c** (B3LYP/6-311+G(2df,2pd))			
	NICS(0)	NICS(0)zz	NICS(1)	NICS(1)zz	NICS(0)	NICS(0)zz	NICS(1)	NICS(1)zz
A	−4.36	4.81	−6.58	−12.80	−2.51	3.53	−5.25	−13.08
B	9.18	52.69	4.18	21.76	10.50	49.86	5.30	20.65
C	3.46	31.00	0.57	9.61	4.82	29.06	1.55	8.96

Ring[a)]	**7** (B3LYP/6-31G(d,p))				**7** (B3LYP/6-311+G(2df,2pd))			
	NICS(0)	NICS(0)zz	NICS(1)	NICS(1)zz	NICS(0)	NICS(0)zz	NICS(1)	NICS(1)zz
A	−5.93	0.37	−8.07	−17.83	−3.93	−0.30	−6.67	−17.55
B	11.22	47.61	3.99	20.06	12.64	46.45	5.09	20.04
C	−5.00	6.47	−7.40	−13.87	−2.70	6.82	−5.70	−12.82
D	−6.22	0.01	−8.74	−18.91	−4.06	0.02	−7.13	−18.18
E	5.21	36.74	0.55	10.60	7.37	37.12	2.09	11.47

Ring[a)]	**CHI1** (B3LYP/6-31G(d,p))				**CHI1** (B3LYP/6-311+G(2df,2pd))			
	NICS(0)	NICS(0)zz	NICS(1)	NICS(1)zz	NICS(0)	NICS(0)zz	NICS(1)	NICS(1)zz
A	−7.43	−3.48	−9.12	−20.85	−5.54	−4.88	−7.89	−21.31
B	1.48	34.48	−1.80	5.25	3.03	32.26	−0.67	4.40
C	1.82	27.29	−1.66	4.04	3.72	26.57	−0.40	4.01
D	−7.49	−3.01	−9.58	−21.28	−5.54	−3.70	−8.14	−21.16
E	−2.48	14.33	−5.62	−7.79	−0.56	13.97	−4.23	−7.41

Ring[a)]	**CHI1’** (B3LYP/6-31G(d,p))				**CHI1’** (B3LYP/6-311+G(2df,2pd))			
	NICS(0)	NICS(0)zz	NICS(1)	NICS(1)zz	NICS(0)	NICS(0)zz	NICS(1)	NICS(1)zz
A	−7.08	−2.96	−8.80	−20.22	−5.11	−4.17	−7.54	−20.55
B	2.80	35.70	−1.29	5.79	4.33	33.06	−0.24	4.56
C	2.71	29.41	−0.99	5.67	4.69	28.79	0.33	5.77
D	−7.56	−3.34	−9.62	−21.69	−5.51	−3.59	−8.10	−21.17
E	−2.29	14.77	−5.44	−7.44	−0.03	15.38	−3.77	−6.17

[a] Ring positions are shown in the following drawings

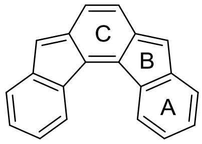

1c

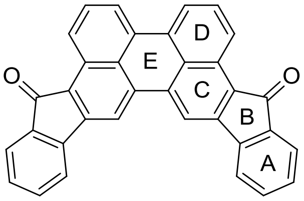

7

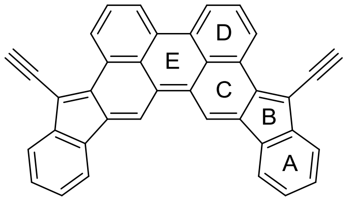

CHI1

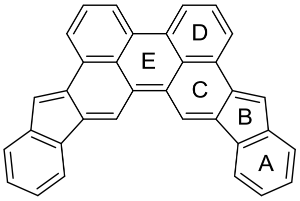

CHI1'

Single point energies with a higher basis set for **CHI1** reveal that the broken symmetry solution has identical energy to that of the restricted closed shell as observed for the **1c** in the literature.[3] Additionally, the square of the spin expectation (spin contamination, $\langle S^2 \rangle$) value was zero for the broken symmetry solution. The broken symmetry solution was tested for stability and found to be stable.

Table S9 Energies of converged wavefunctions for **CHI1**

Method	Total energy (hartree)
RB3LYP/6-311+G(2df,2pd)//B3LYP/6-31G(d,p)	−1459.24708263
BS-UB3LYP/6-311+G(2df,2pd)//B3LYP/6-31G(d,p)	−1459.24708263
UB3LYP/6-311+G(2df,2pd) triplet//B3LYP/6-31G(d,p)	−1459.21657025

The triplet state for **CHI1** is 19.1 kcal mol^{-1} (0.83 eV) above the singlet state.

The singlet biradical character of related compounds was investigated by reoptimizing the geometry using an BS-UB3LYP/6-31G(d,p). The compound **1d** showed the biradical form contributes in the ground-state.

Table S10 Relative energies (in kcal/mol) and spin contamination ($\langle S^2 \rangle$) of **CHI1** and related compounds at the B3LYP/6-31G(d,p) level.

Compd	D*E*(CS – OS)[b]	$\langle S^2 \rangle$[c]	D*E*($T_1 - S_0$)[d]
**1a**	0.00	0.00	26.30
**1b**[4,a]	0.00	0.00	21.65
**1c**	0.00	0.00	23.93
**1d**[5,a]	3.02	0.71	2.76
**CHI1**	0.00	0.00	18.62

[a] The reported optimized structures (B3LYP/6-31G(d)) were used. [b] Closed-shell singlet energy minus open-shell broken symmetry singlet energy. [c] given for S_0. [d] Unrestrected triplet energy minus singlet energy (open-shell singlet for **1d**).

[3] A. G. Fix, P. E. Deal, C. L. Vonnegut, B. D. Rose, L. N. Zakharov, M. M. Haley, *Org. Lett.* **2013**, *15*, 1362.
[4] A. Shimizu, Y. Tobe, *Angew. Chem. Int. Ed.* **2011**, *50*, 6906–6910.
[5] A. Shimizu, R. Kishi, M. Nakano, D. Shiomi, K. Sato, T. Takui, I. Hisaki, M. Miyata, Y. Tobe, *Angew. Chem. Int. Ed.* **2013**, *52*, 6076–6079.

The singlet biradical factor of **CHI1** and related compounds was also calculated by the natural orbital occupation number (NOON) of the LUMO in a spin-unrestricted Hartree-Fock calculation using 6-31G(d) and 6-31G(d,p) basis set.[6] The broken symmetry UHF/6-31G(d,p) calculations gave LUMO occupation number. According to the Yamaguchi scheme,[7] the index for singlet biradical character is expressed as

$$y_i = 1 - \frac{2T_i}{1 + T_i^2}$$

where T_i is the orbital overlap between the corresponding orbital pairs and it can be presented using the NOON of HOMO and LUMO.

$$T_i = \frac{n_{HOMO} - n_{LUMO}}{2}$$

The diradical characters by the theoretical calculation are listed in Table S11.

Table S11 Results on biradical character of **CHI1** and related compounds at the BS-UHF/6-31G(d) and BS-UHF/6-31G(d,p) level[a]

	**1a**	**1b**[4]	**1c**	**1d**[5]	**CHI1**	**CHI1'** [b]	**TIPS-pentacene**[8]	**Pentacene**
n_{LUMO}	0.59	0.61	0.59	0.84	0.71	0.67	0.70	0.68
n_{HOMO}	1.41	1.39	1.41	1.16	1.28	1.33	1.30	1.32
y_i	0.30	0.33	0.29	0.68	0.47	0.40	0.45 (0.15)[c]	0.42

[a] The results for BS-UHF/6-31G(d) and BS-UHF/6-31G(d,p) are identical to four significant figures. [b] A parent structure of **CHI1** (diindeno[1,2-*b*:2',1'-*n*]perylene). [c] the experimentally obtained value is indicated in brackets.

In addition, CASSCF(2,2)/6-31G calculation was performed.

Table S12 Results on biradical character of **CHI1** and related compounds calculated at the CASSCF(2,2)/6-31G

	**1a**	**1b**	**1c**	**1d**	**CHI1**	**CHI1'** [b]
n_{LUMO}	0.141	0.137	0.126	0.443	0.066	0.069
n_{HOMO}	1.859	1.863	1.874	1.557	1.934	0.1931

[6] D. D. hnert, J. Koutecký, *J. Am. Chem. Soc.* **1980**, *102*, 1789–1796.
[7] K. Yamaguchi, *Chem. Phys. Lett.* **1975**, *33*, 330–335.
[8] K. Kamada, K. Ohta, A. Shimizu, T. Kubo, R. Kishi, H. Takahashi, E. Botek, B. Champagne, M. Nakano, *J. Phys. Chem. Lett.* **2010**, *1*, 937–940

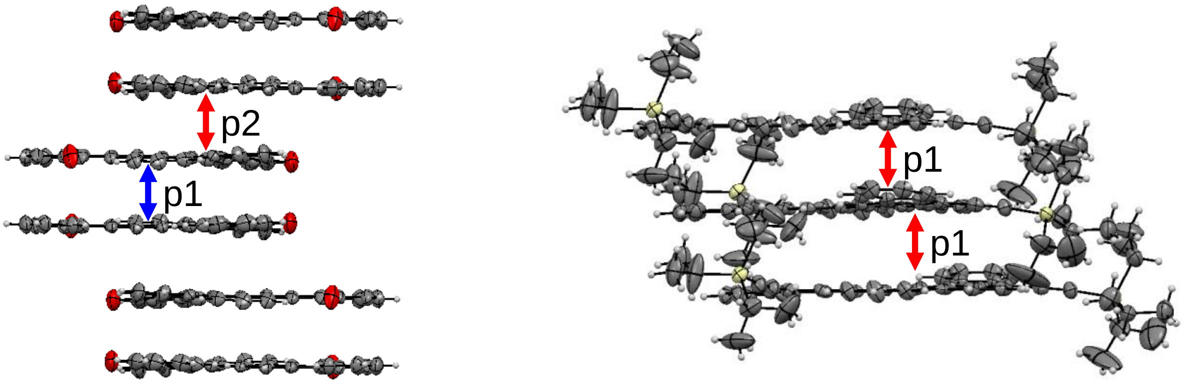

Figure S2. The inter- and intra-dimers of **7** and **CHI1** for which the transfer integral and interactions energy have been calculated at the PW-91/TZP level using the ADF program.

Instruments

Thermogravimetry-differential thermal analysis (TG-DTA) was performed on a TA Instruments SDT Q600 unit under a N_2 atmosphere with a heating rate of 10 °C per minute. X-ray diffraction (XRD) analysis was conducted on a Rigaku Smart Lab Diffractometer operated at 45 kV and 200 mA using CuKα radiation. Atomic force microscopy (AFM) was perfomed on a Bruker Dimension Icon in tapping-mode. Cyclic voltammetry and square wave voltammetry experiments were performed with potentiostat–galvanostat Autolab PGSTAT 12 controlled by GPES software, in a three-electrode single-compartment cell with glassy-carbon or platinum working electrode, a platinum wire counter electrode, and an $Ag/AgNO_3$ reference electrode. The measurements were carried out in dry dichloromethane using a 0.1 M $TBAPF_6$ electrolyte, the solution being purged with argon prior to measurement. All potentials were internally referred to the ferrocene/ferrocenium couple. Square wave voltammetry was performed in the range of potential from −1.8 to 1.5 with scan rate 60 mV/s; step potential, 50 mV; square-wave frequency, 1 Hz; and square-wave amplitude 10 mV.

Synthesis

6,6'-dimethoxy-3,3',4,4'-tetrahydro-1,1'-binaphthalene 1

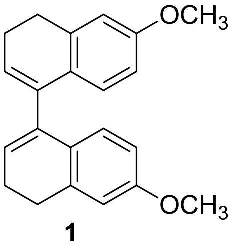

1

310ml of anhydrous THF and 3.2g (48.94mmol) of zinc were added in a three-necked flask under inert atmosphere. This suspension was cooled down to -60°C. Then, 9.92g (56.27mmol) of 6-methoxy-1-tetralone, 24.25ml (191.07 mmol) of TMSCl and 8.3 ml of HCl 37% were added. The solution is left under stirring at -50°C for 30 minutes and then allowed to return to room temperature overnight. Then 100 ml of water and 300 ml of diethyl ether were added. After separation, the organic phase was washed twice with water, dried over Na_2SO_4 and concentrated under reduced pressure. A thick pale brown oil was obtained with a yield of 86% and was used without purification; R_f = 0.30 (eluent: petroleum ether / CH_2Cl_2 ; 70/30); ^{1}H NMR (300 MHz, $CDCl_3$) δ 6.76 (d, *J* = 8.5 Hz, 1H), 6.65 (d, *J* = 2.5 Hz, 1H), 6.46 (dd, *J* = 8.5, 2.7 Hz, 1H), 5.86 (t, *J* = 4.5 Hz, 1H), 3.68 (s, 6H), 2.78 (t, *J* = 7.4 Hz, 2H), 2.29 (td, *J* = 7.9, 4.6 Hz, 2H); ^{13}C NMR (75 MHz, $CDCl_3$) δ 158.33, 138.01, 137.66, 127.96, 126.38, 125.23, 113.57, 110.85, 55.14, 28.79, 23.25.

6,6'-dimethoxy-1,1'-binaphthalene 2

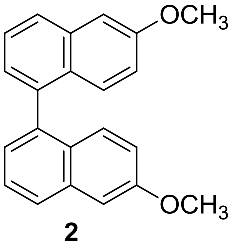

2

3g (9.42mmol) of compound **1** was dissolved in 80mL of anhydrous CH_2Cl_2 in inert atmosphere. The solution was cool down to 0°C then 7.78g (23.55mmol) of trityl fluoroborate was added by small portion. After stirring for 1hat 0°C, 11.82 mL (84.78mmol) of triethylamine was added carefully. The resulting mixture was filtered over a pad of silica gel (CH_2Cl_2) then the filtrate was concentrated under vacuum. The crude product was purified on a silica gel column with petroleum ether and CH_2Cl_2 (70:30) to afford a white solid with a yield of 59%; R_f = 0.35 (eluent: petroleum ether / CH_2Cl_2 ; 70/30); ^{1}H NMR (300 MHz, $CDCl_3$) δ 7.87 (d, *J* = 8.2 Hz, 2H), 7.63 – 7.52 (m, 2H), 7.37 (m, *J* = 12.3, 5.1 Hz, 4H), 7.28 (d, *J* = 2.5 Hz, 2H), 7.00 (dd, *J* = 9.2, 2.6 Hz, 2H), 3.97 (s, 6H); ^{13}C NMR (75 MHz, $CDCl_3$) δ 157.44, 138.49, 134.72, 128.26, 128.13, 126.65, 125.97, 125.55, 118.63, 105.91, 55.28. HRMS (TOF MS ESI$^+$): calculated for $C_{22}H_{18}O_2$ ($M^{\cdot+}$): 314,1307 ; found: 314,1309.

[1,1'-binaphthalene]-6,6'-diol 3

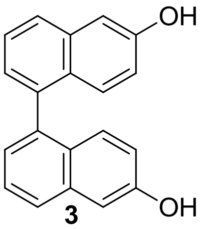

3

1.590g (5.06mmol) of compound **2** was dissolved in 60ml of anhydrous CH_2Cl_2 under inert atmosphere. The mixture was cooled down to 0°C and then 20.23ml (20.23mmol) of boron tribromide solution (1M in hexane) were added dropwise. After 24 hours of stirring, the mixture was hydrolysed carefully at 0°C. The organic phase was washed three times with water, dried over Na_2SO_4 and concentrated under reduced pressure. The crude product was filtered through a pad of silica gel with ethyl acetate and concentrated under vacuum to obtain a pale brown- white solid with a yield of 91%; R_f = 0.05 (CH_2Cl_2) ; ^{1}H NMR (300 MHz, Acetone) δ 8.64 (s, 0.35H), 7.70 (d, *J* = 8.3 Hz, 2H), 7.42 (t, *J* = 7.6 Hz, 2H), 7.21 (d, *J* = 2.5 Hz, 2H), 7.19 – 7.06 (m, 4H), 6.87 (dd, *J* = 9.1, 2.5 Hz, 2H); ^{13}C NMR (75 MHz, Acetone) δ 156.48, 139.90, 136.64, 129.22, 128.96, 127.48, 127.10, 125.93, 119.57, 110.47; HRMS (TOF MS ESI$^+$): calculated for $C_{20}H_{15}O_2$ (MH$^+$): 287.1072 ; found: 287.1069.

Compound 4

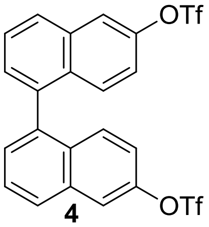

4

3g (10.48mmol) of compound **3** was dissolved in 130ml of anhydrous CH_2Cl_2, then 5.08ml (62.88mmol) of anhydrous pyridine under inert atmosphere. The mixture was cooled down to 0°C and 8.88g (31.44mmol) of trifluoromethanesulfronic anhydride was added dropwise. After 30 min of stirring, the mixture is left to return to romm temperature and stirred for 2 hours. Then, 70mL of water was added and the organic phase was separated, washed three times with water, dried over Na2SO4 and concentrated under reduced pressure. The resulting solution was filtered through a pad of silica gel with petroleum ether and CH_2Cl_2 (50:50) and concentrated under vacuum to obtained a thick pale yellow oil with a yield of 83%; R_f = 0.33 (petroleum ether/CH2Cl2 ; 80/20) ; ^{1}H NMR (300 MHz, $CDCl_3$) δ 7.98 (d, *J* = 8.3 Hz, 2H), 7.84 (d, *J* = 2.5 Hz, 2H), 7.72 – 7.63 (m, 2H), 7.53 (dd, *J* = 7.0, 1.0 Hz, 2H), 7.41 (d, *J* = 9.3 Hz, 2H), 7.18 (dd, *J* = 9.3, 2.5 Hz, 2H);13C NMR (75 MHz, CDCl3): δ = 147.20, 137.72, 133.69, 131.63, 129.17, 129.14, 128.47, 127.24, 119.92, 119.55, 118.76 (CF3, *J* = 318.7 Hz); HRMS (TOF MS ESI $^+$): calculated for $C_{22}H_{12}O_6F_6$S2 ($M^{\cdot+}$): 549.9980 ; found: 549.9971.

Compound 5

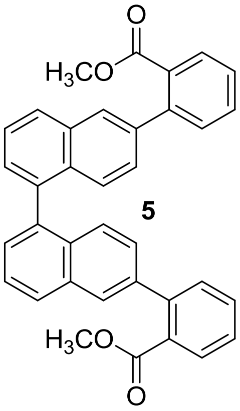

3.5g (6.63mmol) of compound **4**, 6.71g (31.6mmol) of K_3PO_4, 3.58g (19.9mmol) of (2-(methoxycarbonyl)phenyl)boronic acid were weighted together then 60mL of anhydrous DMF was added. The resulting mixture was degased with bubbling argon for 10min then 2.29g (1.98mmol) of $Pd(PPh_3)_4$ was added under inert atmosphere. The solution was heated up at 95°C for 18h. After leaving the solution to return to room temperature, 250ml of water was added. The resulting solid was filtered and washed with water in order to remove DMF, then the product was taking up in CH_2Cl_2 and water was added. The organic layer was separated, washed three times with water, dried over Na_2SO_4, filtered and concentrated under reduced pressure. The resulting solution was filtered through a pad of silica gel then concentrated. The crude product was purified on a silica gel column with petroleum ether and CH_2Cl_2 (50:50) until the subproducts were removed, then was accelerated using a proportion of 30:70 and concentrated under vacuum to give a white solid with a yield of 68%; R_f = 0.49 (petroleum ether/CH_2Cl_2 ; 30/70); ^{1}H NMR (300 MHz, $CDCl_3$) δ 7.99 (d, *J* = 8.1 Hz, 2H), 7.92 (s, 2H), 7.88 (d, *J* = 7.7 Hz, 2H), 7.70 – 7.40 (m, 12H), 7.27 (dd, *J* = 9.3, 2.5 Hz, 2H), 3.62 (s, 6H); ^{13}C NMR (75 MHz, $CDCl_3$) δ 169.02, 142.26, 138.84, 138.20, 133.41, 131.82, 131.40, 131.07, 130.83, 129.93, 128.14, 128.06, 127.30, 127.08, 126.07, 125.75, 51.99; HRMS (TOF MS ESI +): calculated for $C_{36}H_{27}O_4$ (MH^+): 535.1909 ; found: 523.1902.

11H,11'H-[4,4'-bibenzo[a]fluorene]-11,11'-dione 6

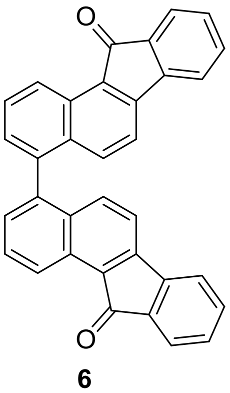

6

1.10g (2.10mmol) of **5** was added under inert atmosphere to a flask containing 125 ml of methanesulfonic acid. The resulting suspension was heated at 75°C. After 3 hours of stirring, the reaction was quenched by adding carefully 350ml of cold water in order to induce precipitation. A yellow solid was obtained and filtered, washed with water then with petroleum ether. The residual solvent was evaporated under vacuum to obtain a yellow solid sparkly soluble in common solvents with a yield of 97%; R_f = 0.27; ^{1}H NMR (300 MHz, Pyr): δ 9.40 (d, *J* = 8.5 Hz, 2H), 7.80 – 7.65 (m, 8H), 7.52 (t, *J* = 7.0 Hz, 4H), 7.41 (t, *J* = 7.3 Hz, 2H), 7.28 (t, *J* = 7.4 Hz, 2H); ^{13}C NMR (75 MHz, Pyr): δ 135.33 (CH), 135.21 (CH), 130.36 (CH), 129.86 (CH), 129.16 (CH), 124.95 (CH), 124.47 (CH), 121.43 (CH), 119.67 (CH), quaternary carbons did not show up for solubility reasons; HRMS (TOF MS ESI $^+$): calculated for $C_{34}H_{19}O_2$ ($M^{\cdot +}$): 459.1385 ; found: 459.1384; IR: 3044, 1696, 1601, 1579, 1522, 1464, 1411, 1363, 1294, 1178, 1152, 1099, 1058, 924, 945, 843, 826, 808, 771, 749, 712 cm^{-1}.

Diindeno[1,2-b:2',1'-n]perylene-5,12-dione 7

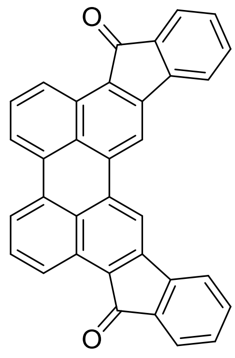

7

964mg (2.10mmol) of compound **6** and 7.4g (52.5mmol) of K_2CO_3 were added to 43.5ml of 2-aminoethanol under inert atmosphere. The resulting mixture was heated up at 160°C. After two hours of stirring, the dark blue solution was put in a 1L Erlenmeyer flask with the help of water then 800ml in total of water was added. The resulting blue precipitate was filtered, washed with water and then with petroleum ether. The blue product was dried under reduced pressure and obtained with a yield of 95%; IR: 3045, 1691, 1605, 1580, 1530, 1464, 1411, 1354, 1283, 1213, 1175, 1149, 1118, 1081, 1050, 930, 888, 863, 820, 783, 763 cm^{-1}; UV/Vis (toluene): λ^{max} =592, 556, 455, 430 nm; elemental analysis calcd (%) for $C_{34}H_{16}O_2$: C 89.46, H 3.53; found: C 89.55, H 3.52.

12-bis((triisopropylsilyl)ethynyl)-5,12-dihydrodiindeno[1,2-b:2',1'-n]perylene-5,12-diol 8

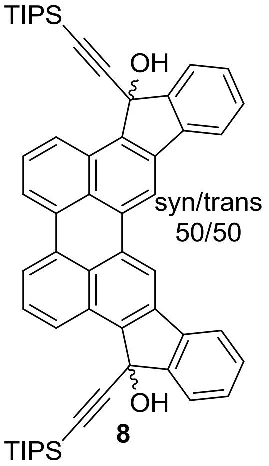

8

110mg (0.241mmol) of compound **7** was put in suspension in 125ml of anhydrous THF under inert atmosphere. The solution was ultrasonicated for 1h at room temperature, then the mixture was cooled down to 0°C. 6.02mmol (25eq.) of ((triisopropylsilyl)ethynyl)lithium (prepared classically from triisopropylsilylacetylene and *n*-buLi) was transferred via a cannula. The reaction was instantaneous. Then 100ml of a supersaturated aqueous solution of NH_4Cl were added followed by the addition of 200 ml of diethyl ether. The organic layer was separated, washed twice with water, dried over $MgSO_4$ and concentrated under reduced pressure. The crude product was purified on a silica gel column using hexane and CH_2Cl_2 (in proportion of 30:70 in the beginning, then accelerated to 100% of CH_2Cl_2) and concentrated under vacuum to obtain two diastereoisomers with different polarity in a yield of 88%; First diastereosiomer : R_f = 0.39 (hexane/CH_2Cl_2 , 25/75); ^{1}H NMR (300 MHz, $CDCl_3$) δ 8.36 (d, *J* = 7.9 Hz, 2H), 8.02 (s, 2H), 7.82 (d, *J* = 6.9 Hz, 2H), 7.69 (d, *J* = 6.6 Hz, 2H), 7.40 – 7.16 (m, 8H), 0.99 (s, 42H); ^{13}C NMR (75 MHz, $CDCl_3$) δ 148.22, 140.38, 138.89, 137.22, 133.10, 131.58, 129.93, 129.39, 128.76, 128.33, 127.15, 124.71, 123.54, 120.23, 120.06, 112.62, 107.08, 86.03, 75.40, 18.61, 11.19. HRMS (TOF MS ESI $^+$): calculated for $C_{56}H_{60}O_2Si_2$ ($M^{\cdot+}$): 820.4132 ; found: 820.4123. Second diastereosiomer: R_f = 0.09 (hexane/CH_2Cl_2 , 25/75); ^{1}H NMR (300 MHz, $CDCl_3$) δ 8.36 (d, *J* = 7.9 Hz, 2H), 8.29 (s, 2H), 8.02 (d, *J* = 6.6 Hz, 2H), 7.73-7.67 (m, 4H), 7.47 – 7.34 (m, 6H), 0.98 (s, 42H); ^{13}C NMR (75 MHz, $CDCl_3$) δ 148.38, 140.41, 139.08, 137.04, 133.19, 131.60, 129.99, 129.37, 128.85, 128.36, 127.21, 124.67, 123.72, 120.32, 119.93, 112.45, 106.89, 85.96, 75.34, 18.54, 11.10; HRMS (TOF MS ESI $^+$): calculated for $C_{56}H_{60}O_2Si_2$ ($M^{\cdot+}$): 820.4132 ; found: 820.4122.

5,12-bis((triisopropylsilyl)ethynyl)diindeno[1,2-b:2',1'-n]perylene CHI1

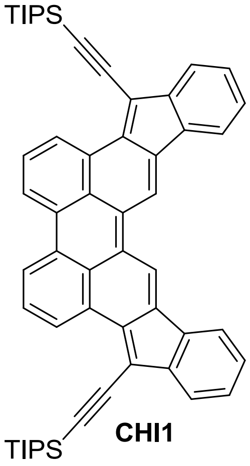

CHI1

110mg (0.241mmol) of compound **7** was put in suspension in 125ml of anhydrous THF under inert atmosphere. The solution was ultrasonicated for 1h at room temperature, then the mixture was cooled at 0°C. 6.02mmol (25eq.) of ((triisopropylsilyl)ethynyl)lithium (prepared classically from triisopropylsilylacetylene and *n*-buLi) was transferred via a cannula. The reaction was instantaneous. Then 100ml of a supersaturated aqueous solution of NH_4Cl were added followed by the addition of 200 ml of diethyl ether. The organic layer was separated, washed twice with water, dried over $MgSO_4$ and concentrated under reduced pressure. The crude product obtained was dried overnight under reduced pressure. The crude product was dissolved in 25ml of anhydrous toluene, then 183mg (0.964mmol) of anhydrous SnCl2 was added. The solution was heated at 120°C and after one hour of stirring under inert atmosphere, the mixture was filtered through a pad of silica gel with toluene and hexane (50:50) and concentrated under vacuum to obtain a blue solid with a yield of 63%; Rf = 0.71 (hexane/toluene , 50/50); ^{1}H NMR (200 MHz, C_6D_6) δ 9.53 (d, *J* = 7.8 Hz, 2H), 8.12 (d, *J* = 7.6 Hz, 2H), 7.96 (d, *J* = 6.1 Hz, 2H), 7.88 (s, 2H), 7.67 (d, *J* = 6.7 Hz, 2H), 7.58 – 7.42 (m, 8H), 1.62 (s, 6H), 1.60 (s, 36H). IR (cm^{-1}): 3050, 2939, 2862, 2111, 1587, 1461, 1443, 1334, 1053, 1014, 993, 879, 800, 749, 700; UV/Vis (toluene): λ^{max} (ε $mol^{-1}cmL^{-1}$)=683 (71000), 636 (72400), 541 (48900), 498 (47600), 466 (36800), 442 (30700) nm;elemental analysis calcd (%) for $C_{56}H_{58}Si_2$: C 85.44, H 7.43; found: C 85.55, H 7.42.

X-ray structures

11H,11'H-[4,4'-dibenzo[a]fluorene]-11,11'-dione 6

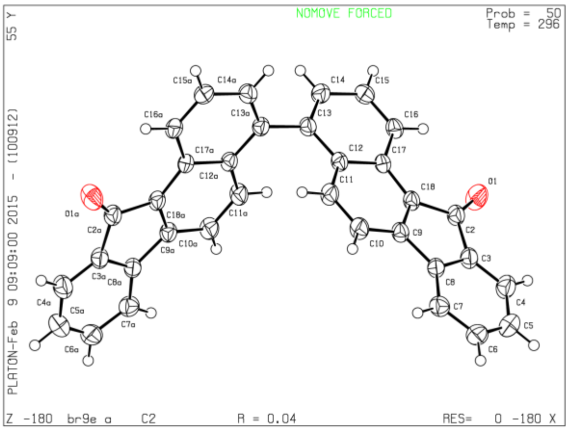

Datablock: **6**
Bond precision: C-C = 0.0024 A Wavelength=0.71073
Cell: a=13.707(2) b=6.3468(10) c=13.269(3)
alpha=90 beta=103.472(8) gamma=90
Temperature: 296 K

	Calculated	Reported
Volume	1122.6(4)	1122.5(3)
Space group	C 2	C2
Hall group	C 2y ?	
Moiety formula	C34 H18 O2 ?	
Sum formula	C34 H18 O2	C34 H18 O2
Mr	458.48	458.48
Dx,g cm-3	1.356	1.356
Z	2	2
Mu (mm-1)	0.083	0.083
F000	476.0	476.0
F000’	476.21	
h,k,lmax	19,8,18	19,8,18
Nref	3330[1802]	2849
Tmin,Tmax	0.980,0.990	0.980,0.990
Tmin’	0.980	

Correction method= # Reported T Limits: Tmin=0.980 Tmax=0.990
AbsCorr = NONE
Data completeness= 1.58/0.86 Theta(max)= 30.170
R(reflections)= 0.0402(2475) wR2(reflections)= 0.1215(2849)
S = 1.121 Npar= 165

Diindeno[1,2-b:2',1'-n]perylene-5,12-dione 7

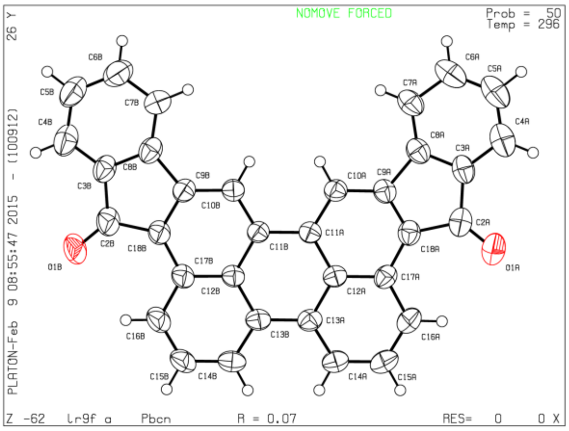

Datablock: 7
Bond precision: C-C = 0.0040 A Wavelength=0.71073
Cell: a=18.9562(15) b=15.4930(15) c=14.8168(14)
alpha=90 beta=90 gamma=90
Temperature: 296 K

	Calculated	Reported
Volume	4351.5(7)	4351.5(7)
Space group	P b c n	Pbcn
Hall group	-P 2n 2ab ?	
Moiety formula	C34 H16 O2 ?	
Sum formula	C34 H16 O2	C34 H16 O2
Mr	456.47	456.47
Dx,g cm -3	1.393	1.394
Z	8	8
Mu (mm-1)	0.086	0.086
F000	1888.0	1888.0
F000’	1888.83	
h,k,lmax	22,18,17	22,18,17
Nref	3843	3842
Tmin,Tmax	0.994,0.997	0.983,0.997
Tmin’	0.983	

Correction method= # Reported T Limits: Tmin=0.983 Tmax=0.997
AbsCorr = MULTI-SCAN
Data completeness= 1.000 Theta(max)= 25.000
R(reflections)= 0.0651(2051) wR2(reflections)= 0.1900(3842)
S = 0.946 Npar= 326

5,12-bis((triisopropylsilyl)ethynyl)diindeno[1,2-b:2',1'-n]perylene CHI1

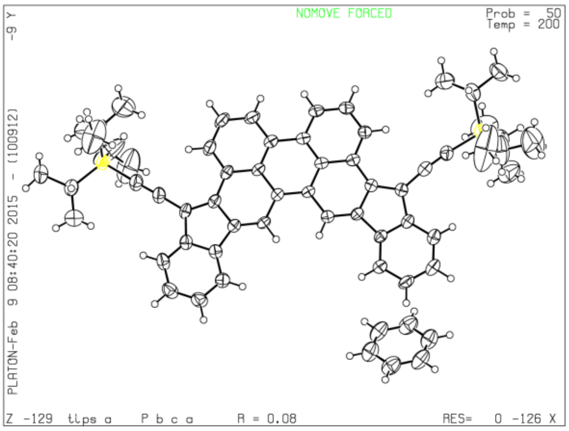

Datablock: CHI1

Bond precision: C-C = 0.0062 A Wavelength=0.71073

Cell: a=34.006(2) b=7.6167(5) c=36.832(2)

alpha=90 beta=90 gamma=90

Temperature: 200 K

	Calculated	Reported
Volume	9540.0(10)	9540.0(10)
Space group	P b c a	P b c a
Hall group	-P 2ac 2ab	-P 2ac 2ab
Moiety formula	C56 H58 Si2, 0.5(C6 H6)	?
Sum formula	C59 H61 Si2	C59 H61 Si2
Mr	826.26	826.25
Dx,g cm-3	1.151	1.151
Z	8	8
Mu (mm-1)	0.112	0.112
F000	3544.0	3544.0
F000’	3546.43	
h,k,lmax	40,9,43	40,9,43
Nref	8463	8431
Tmin,Tmax	0.984,0.996	
Tmin’	0.967	

Correction method= Not given

Data completeness= 0.996 Theta(max)= 25.082

R(reflections)= 0.0778(4355) wR2(reflections)= 0.2072(8431)

S = 1.022 Npar= 562

NMR Spectra

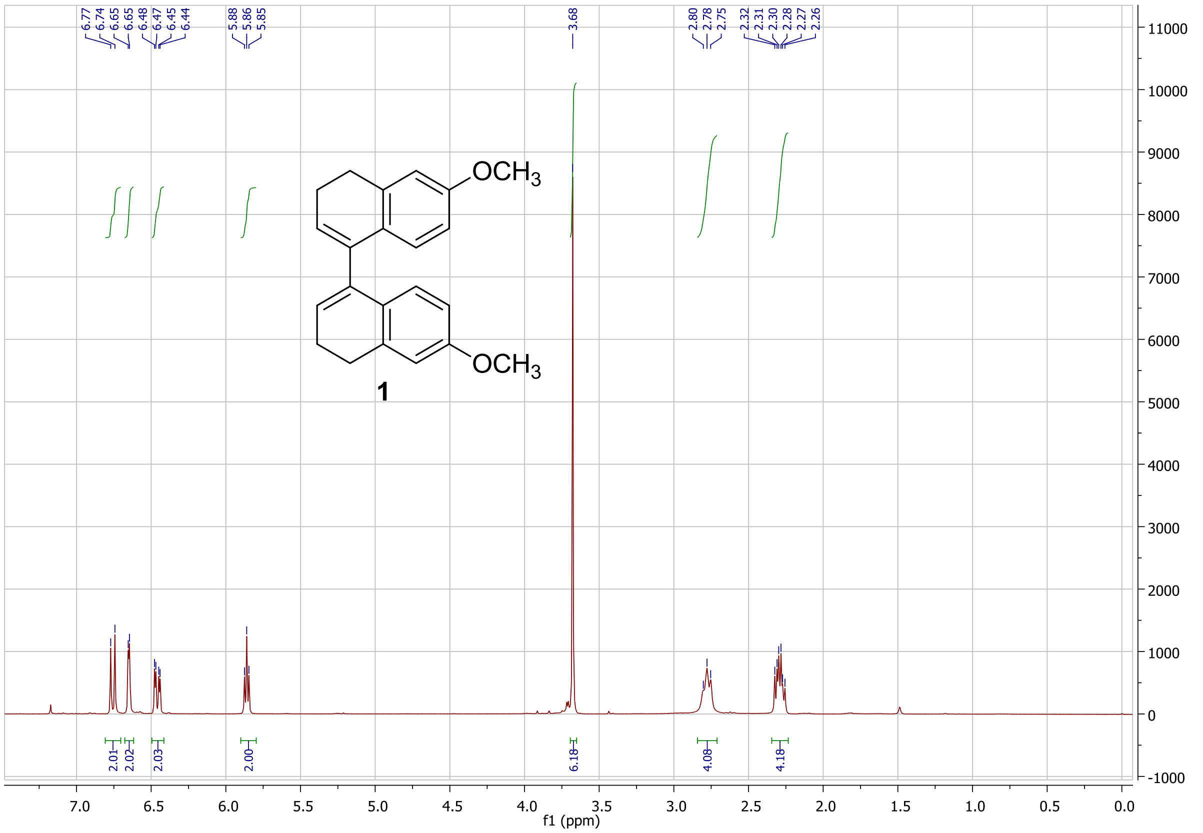
6.77
6.74
6.65
6.65
6.48
6.47
6.45
6.44
5.88
5.86
5.85
3.68
2.80
2.78
2.75
2.32
2.31
2.30
2.28
2.27
2.26
OCH3
OCH3
1
2.01
2.02
2.03
2.00
6.18
4.08
4.18
f1 (ppm)

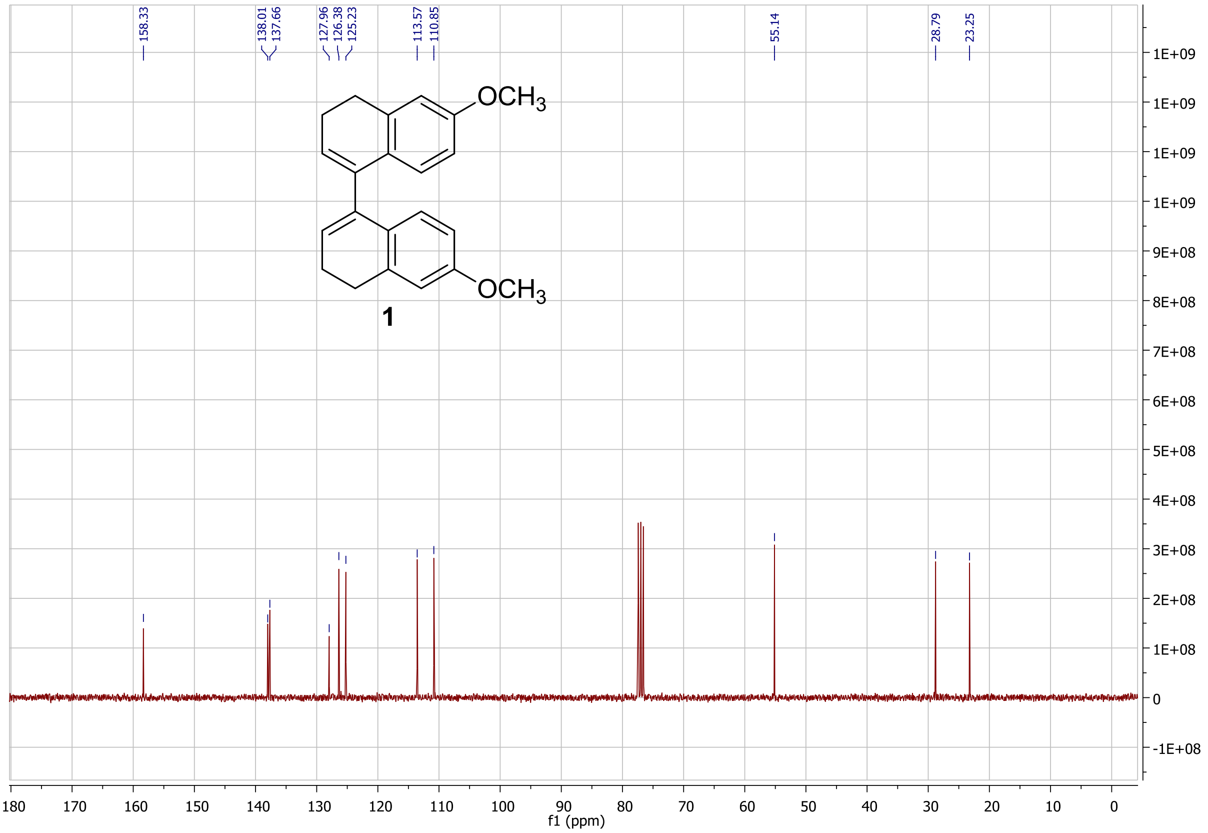
158.33
138.01
137.66
127.96
126.38
125.23
113.57
110.85
55.14
28.79
23.25
OCH3
OCH3
1
f1 (ppm)

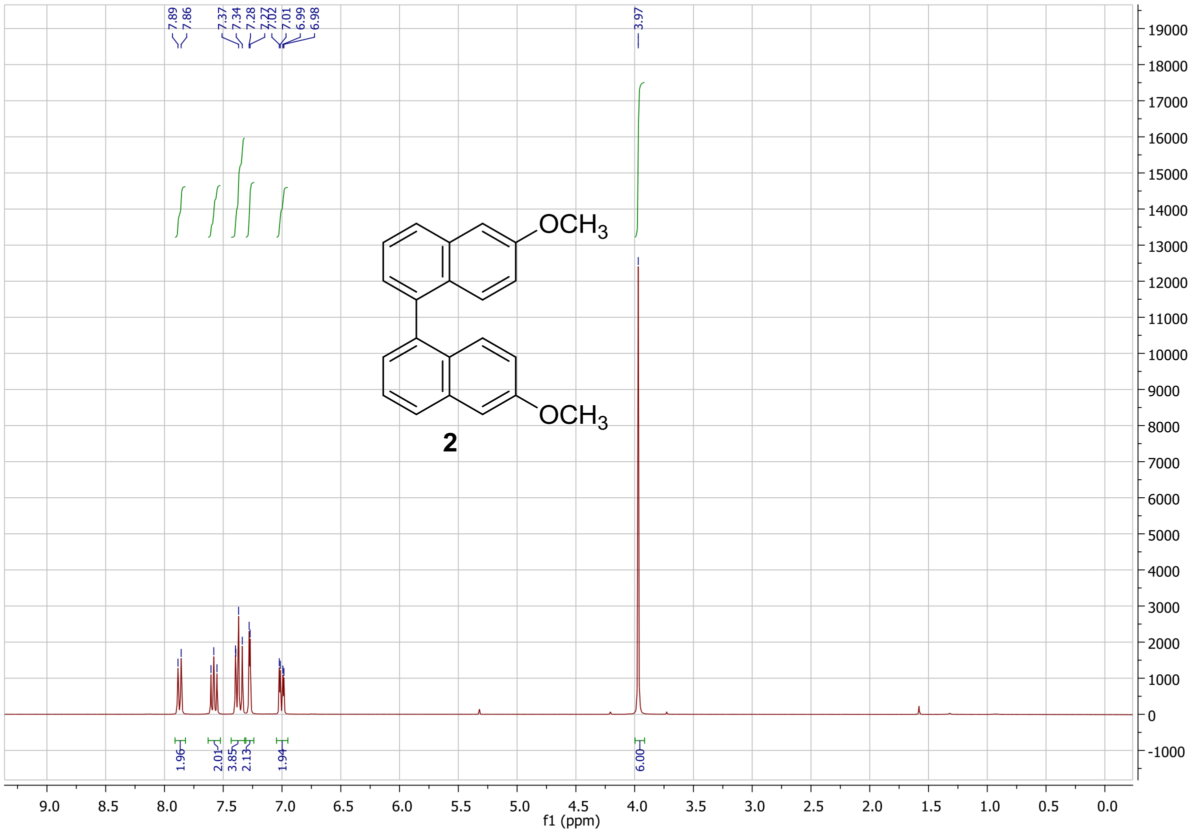

7.89
7.86
7.37
7.34
7.28
7.27
7.02
7.01
6.99
6.98
3.97
OCH3
OCH3
2
1.96
2.01
3.85
2.13
1.94
6.00
f1 (ppm)

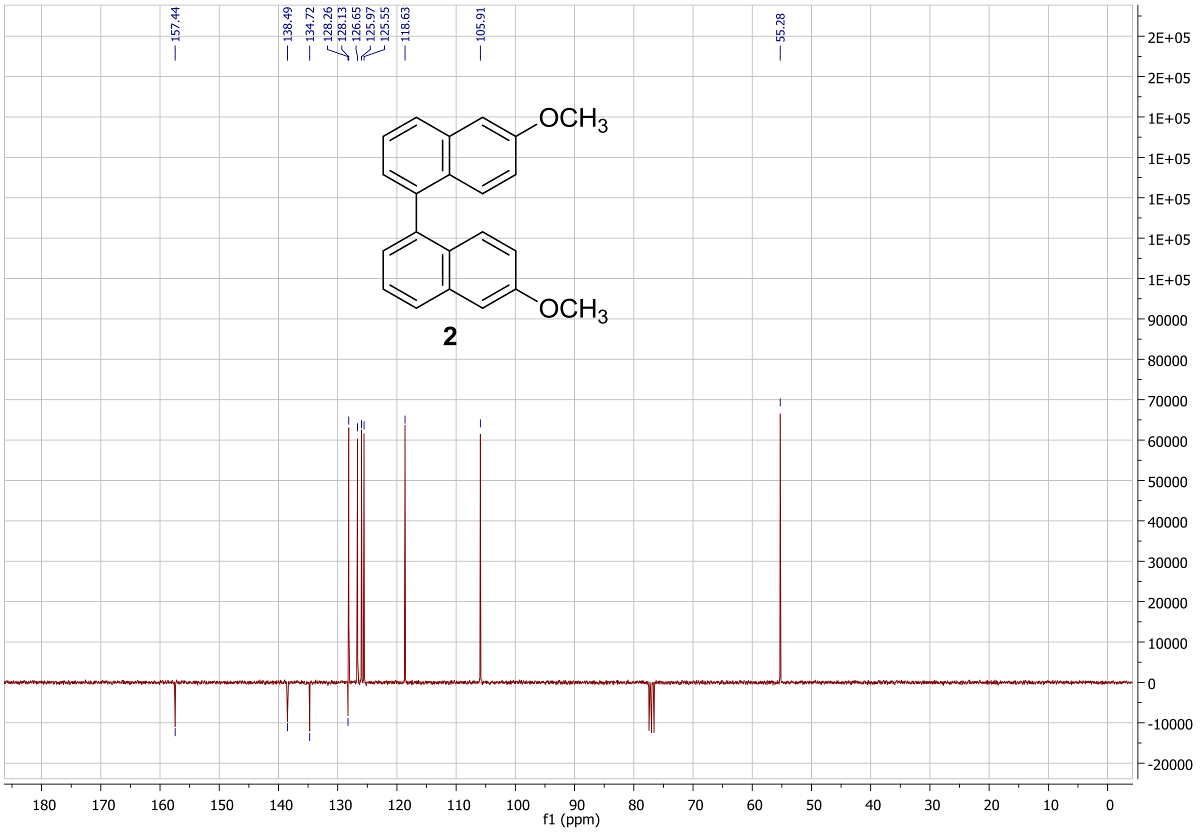

157.44
138.49
134.72
128.26
128.13
126.65
125.97
125.55
118.63
105.91
55.28
OCH3
OCH3
2
f1 (ppm)

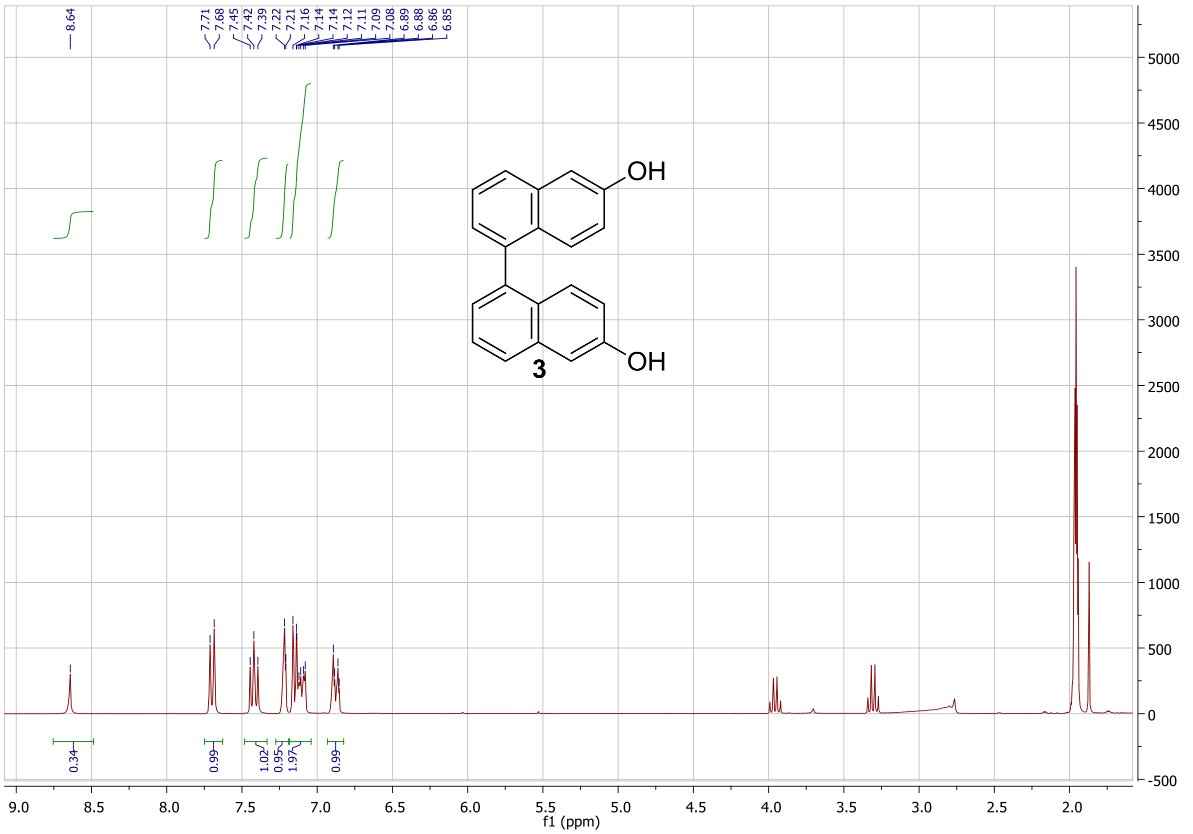
8.64
7.71
7.68
7.45
7.42
7.39
7.22
7.21
7.16
7.14
7.14
7.12
7.11
7.09
7.08
6.89
6.88
6.86
6.85
OH
OH
3
0.34
0.99
1.02
0.95
1.97
0.99
f1 (ppm)

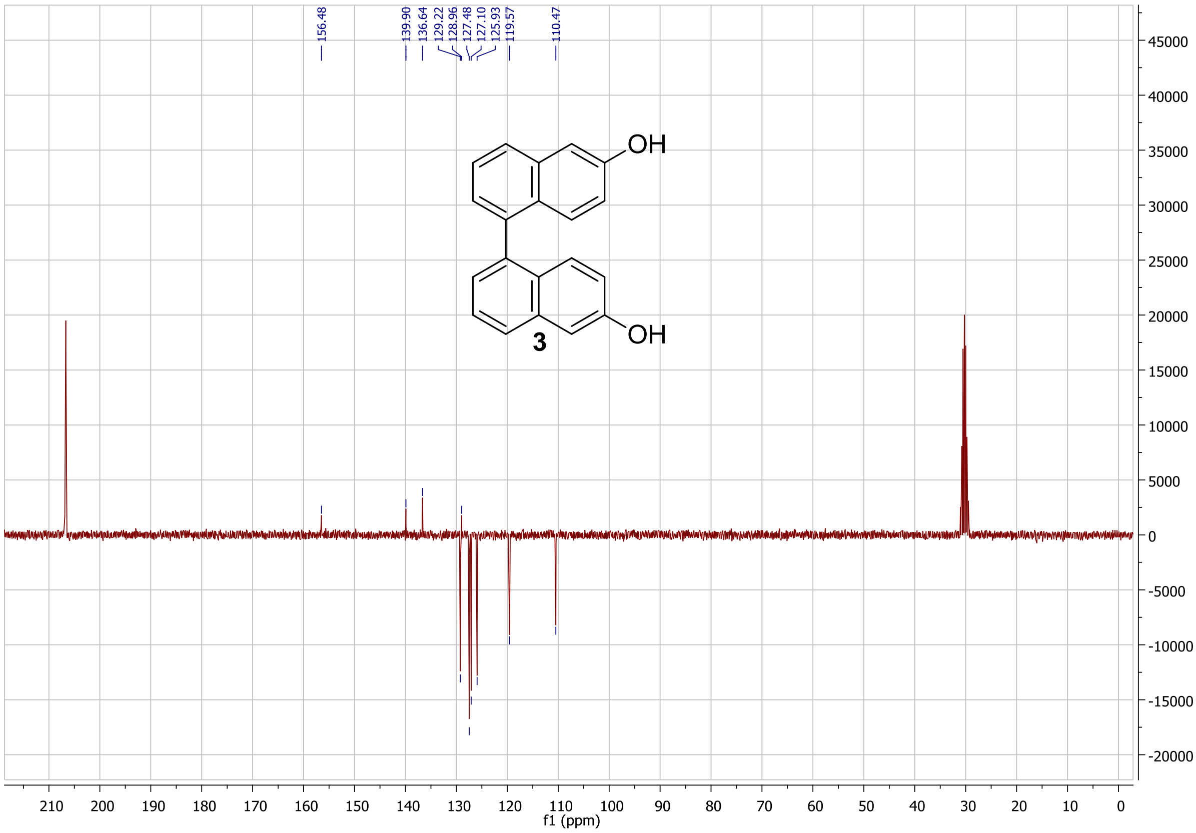
156.48
139.90
136.64
129.22
128.96
127.48
127.10
125.93
119.57
110.47
OH
OH
3
f1 (ppm)

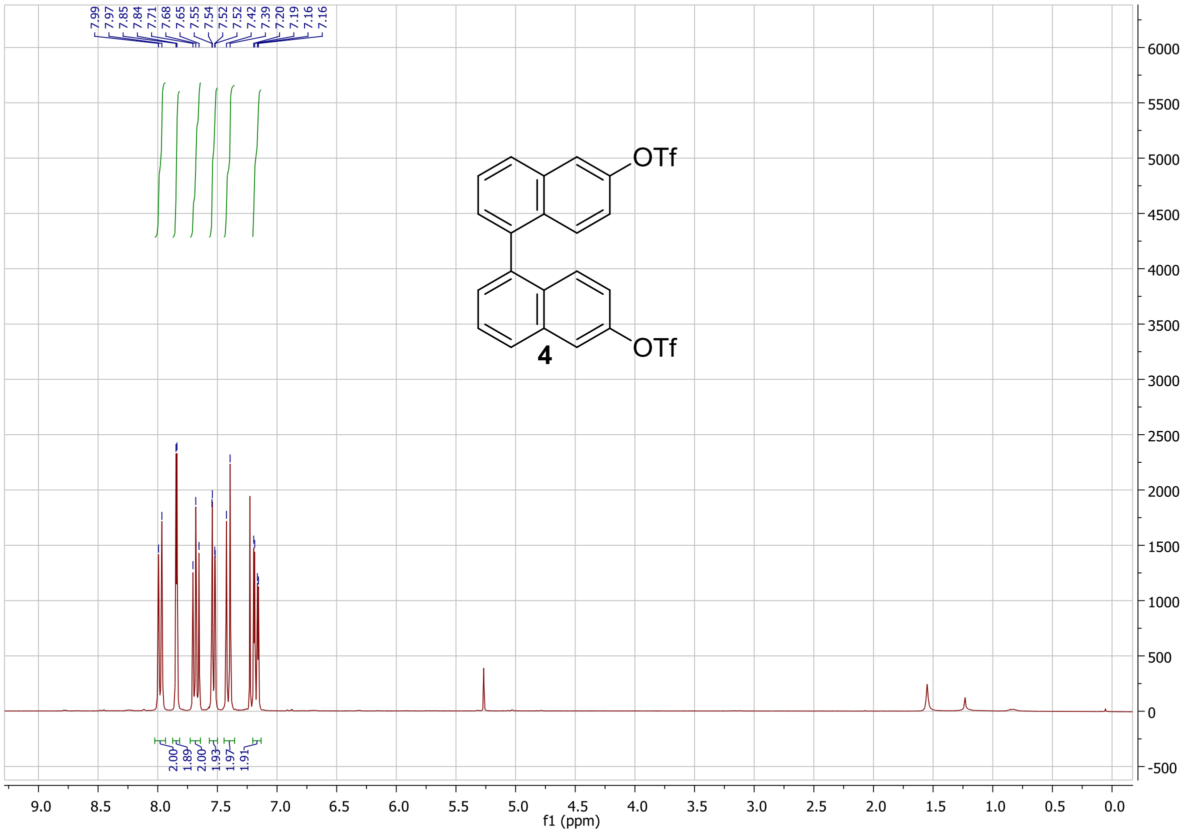
7.99
7.97
7.85
7.84
7.71
7.68
7.65
7.55
7.54
7.52
7.52
7.42
7.39
7.20
7.19
7.16
7.16
OTf
OTf
4
2.00
1.89
2.00
1.93
1.97
1.91
f1 (ppm)

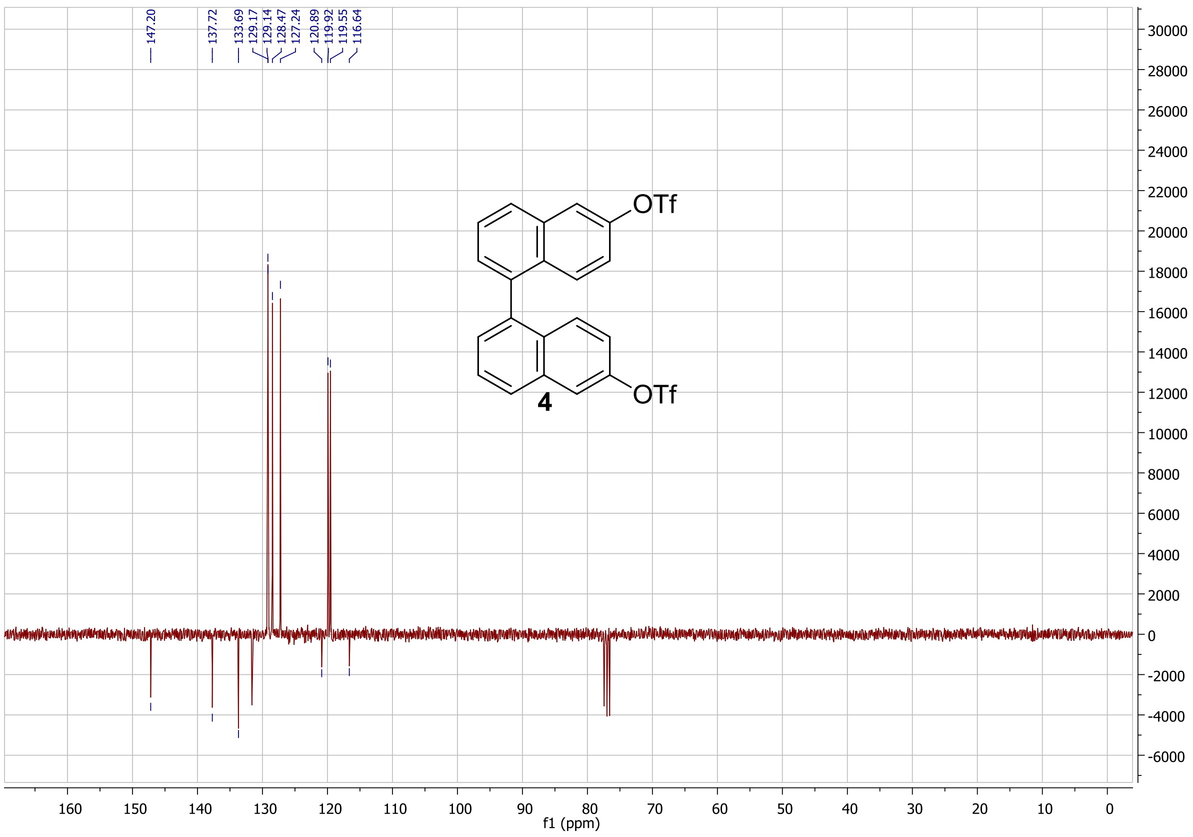
147.20
137.72
133.69
129.17
129.14
128.47
127.24
120.89
119.92
119.55
116.64
OTf
OTf
4
f1 (ppm)

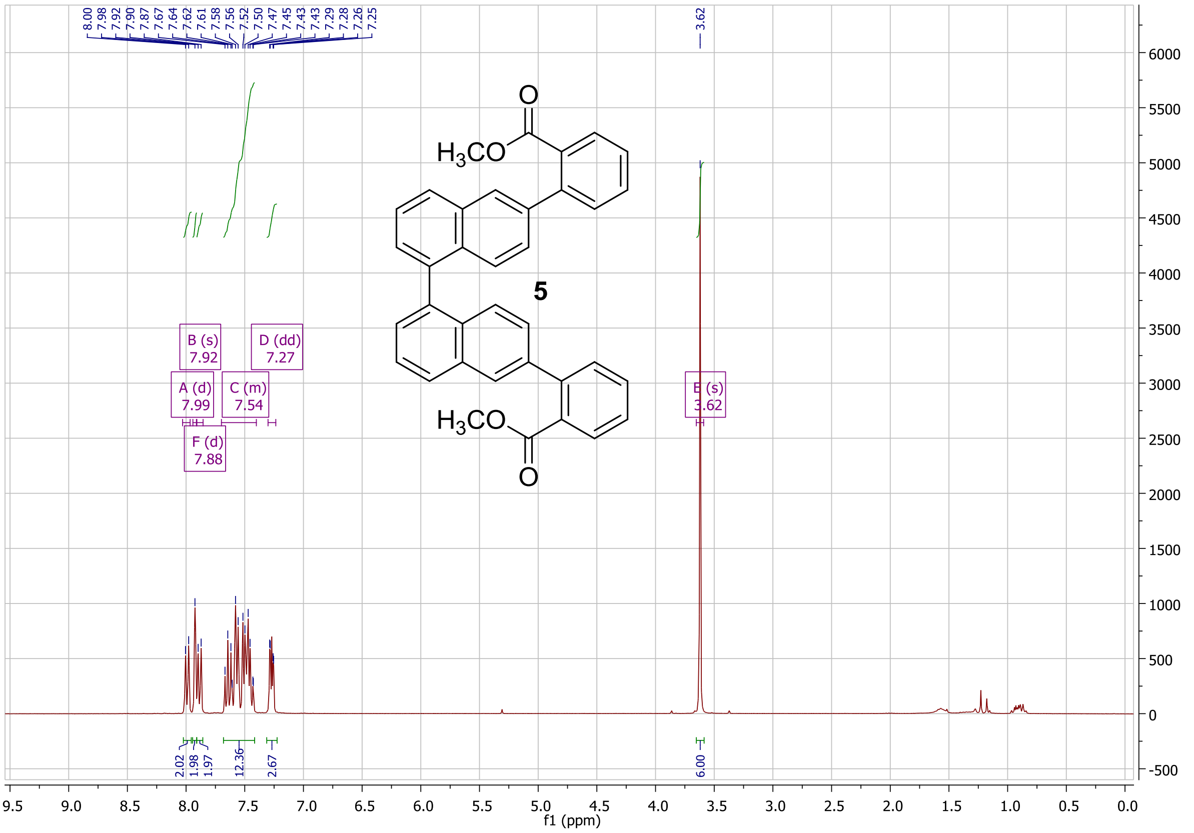
8.00
7.98
7.92
7.90
7.87
7.67
7.64
7.62
7.61
7.58
7.56
7.52
7.50
7.47
7.45
7.43
7.43
7.29
7.28
7.26
7.25
3.62
B (s)
7.92
D (dd)
7.27
A (d)
7.99
C (m)
7.54
F (d)
7.88
E (s)
3.62
5
2.02
1.98
1.97
12.36
2.67
6.00
f1 (ppm)

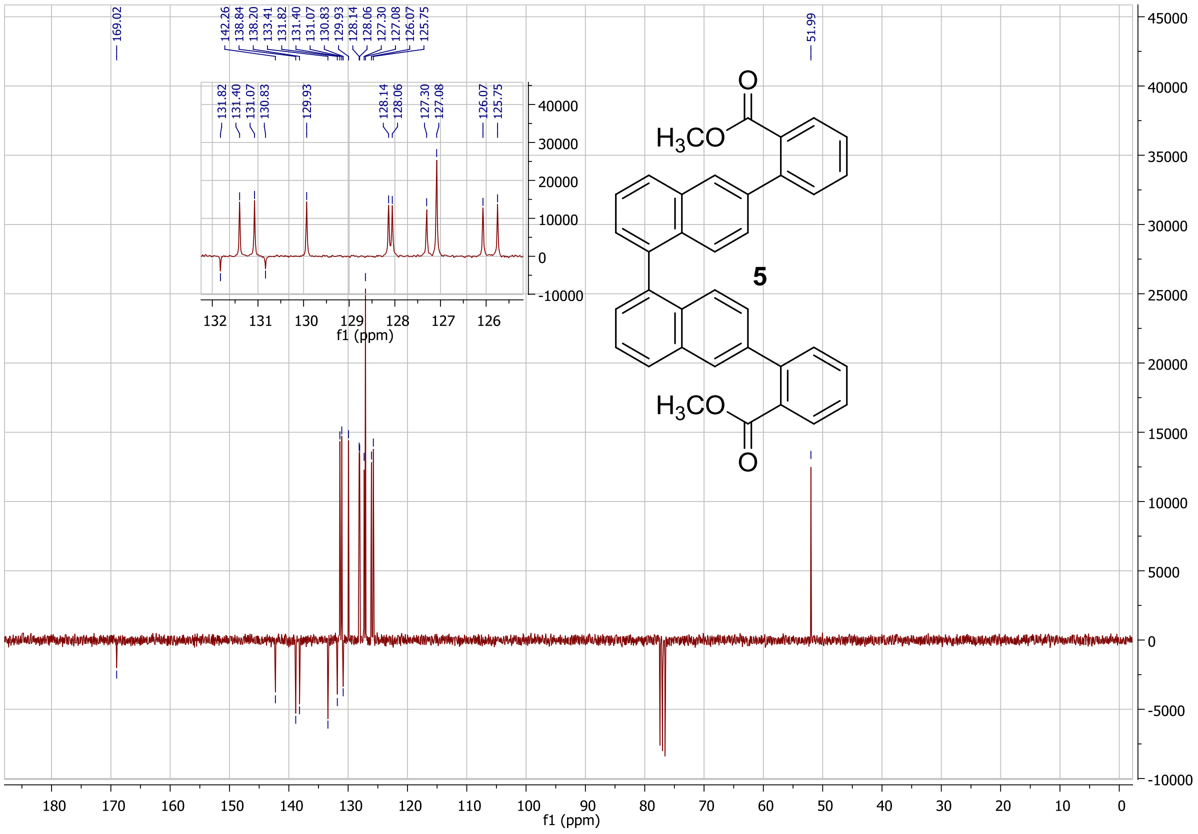
169.02
142.26
138.84
138.20
133.41
131.82
131.40
131.07
130.83
129.93
128.14
128.06
127.30
127.08
126.07
125.75
51.99
5
f1 (ppm)

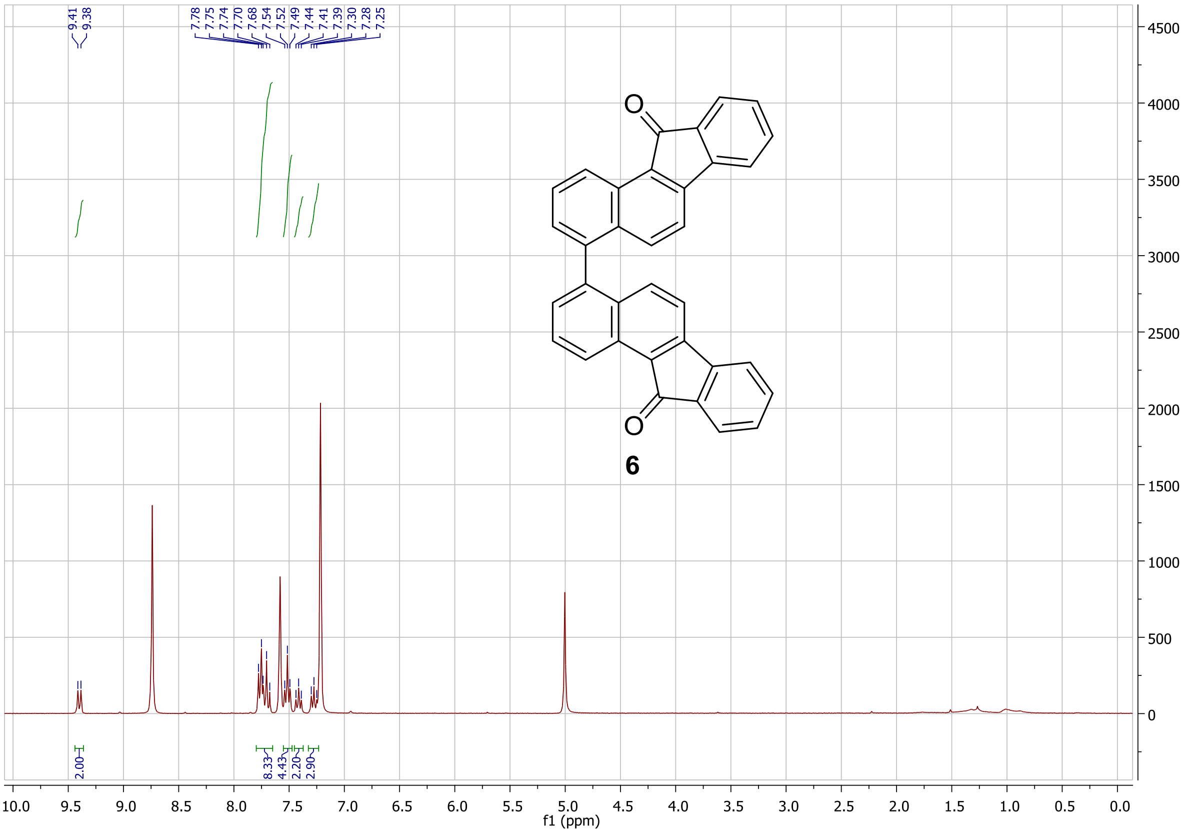
9.41
9.38
7.78
7.75
7.74
7.70
7.68
7.54
7.52
7.49
7.44
7.41
7.39
7.30
7.28
7.25
2.00
8.33
4.43
2.20
2.90
6
f1 (ppm)

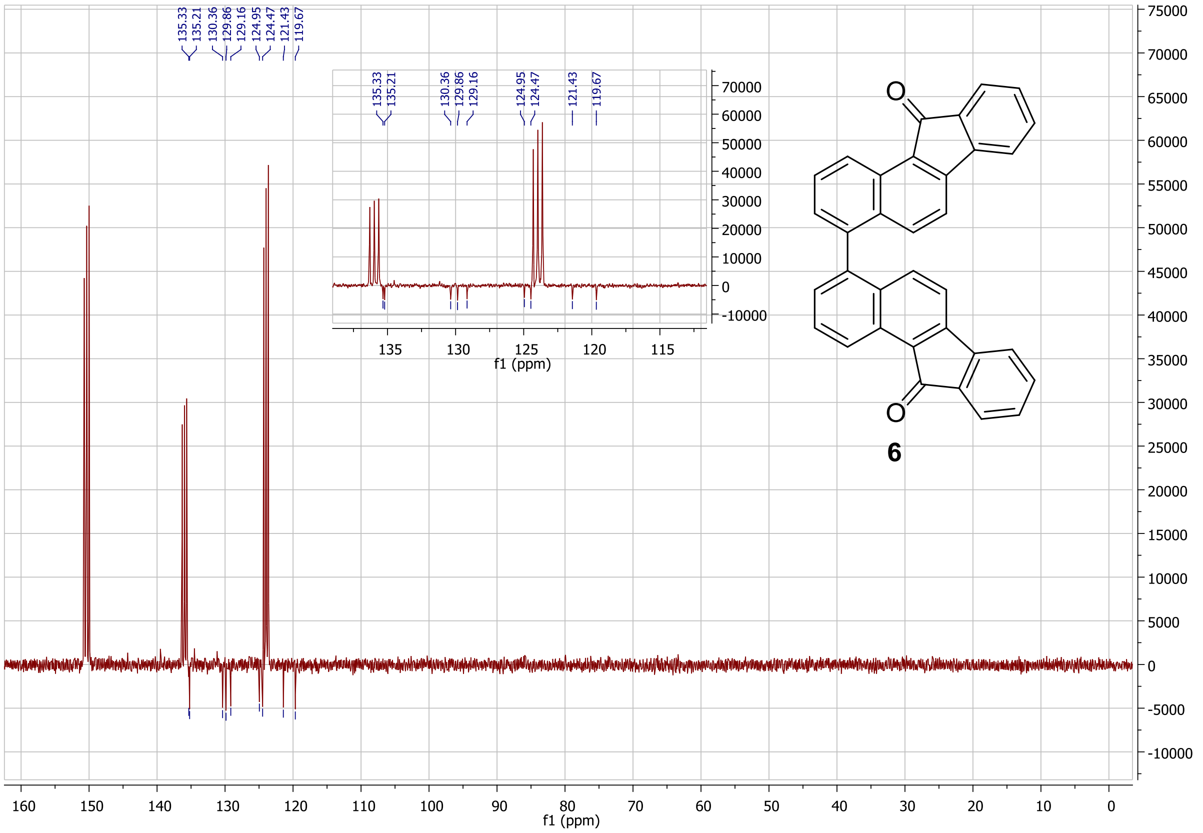
135.33
135.21
130.36
129.86
129.16
124.95
124.47
121.43
119.67
6
f1 (ppm)

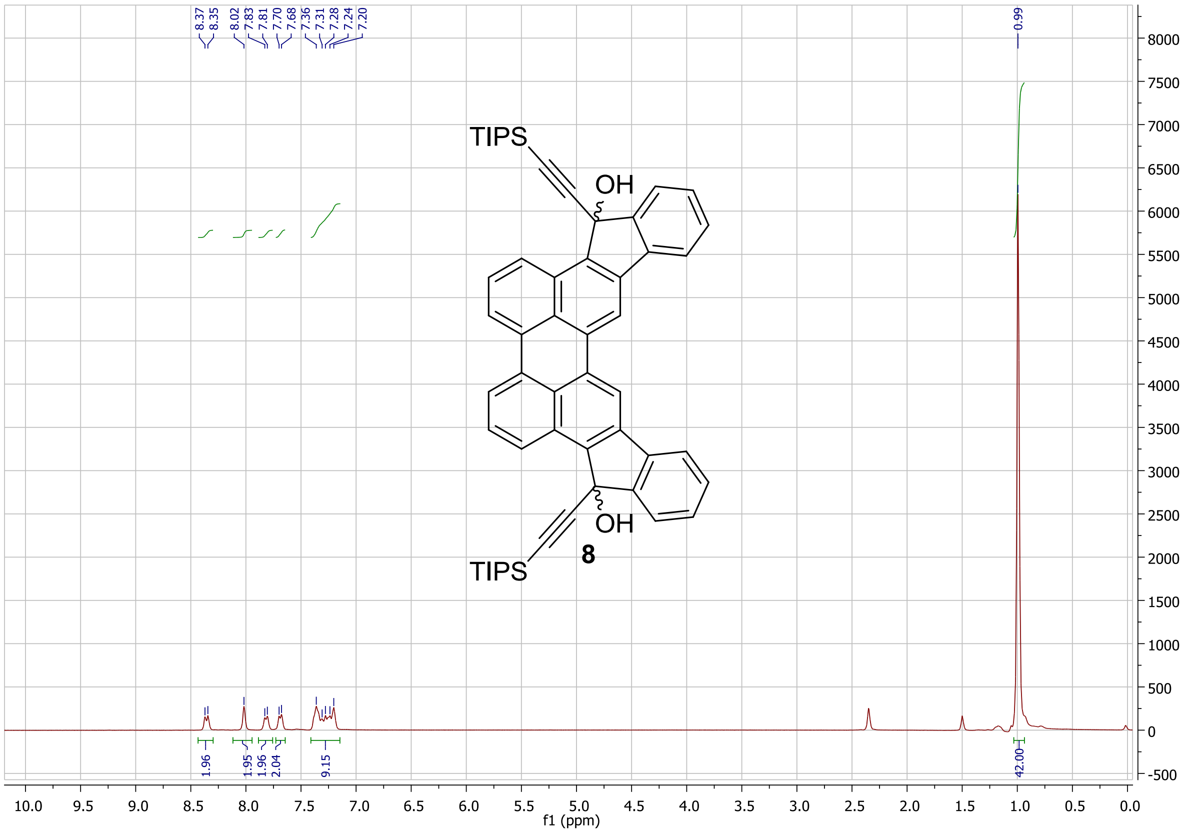

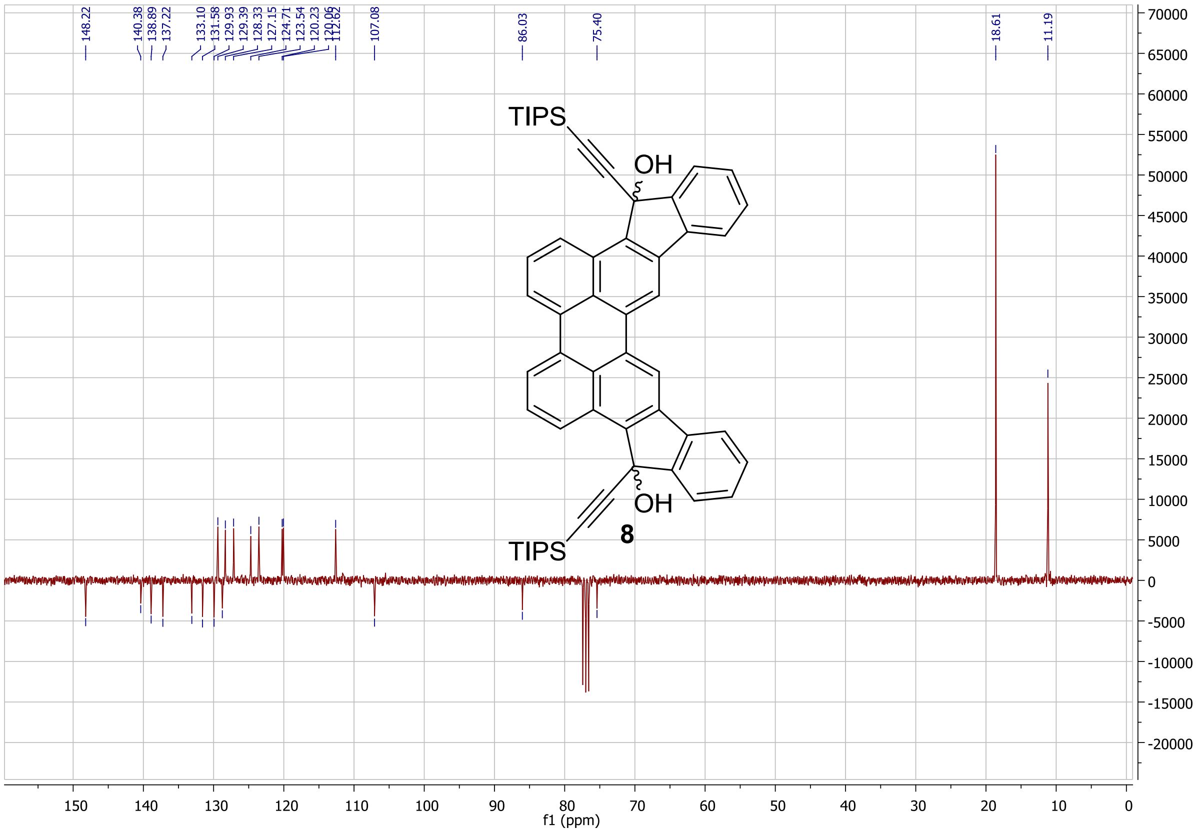

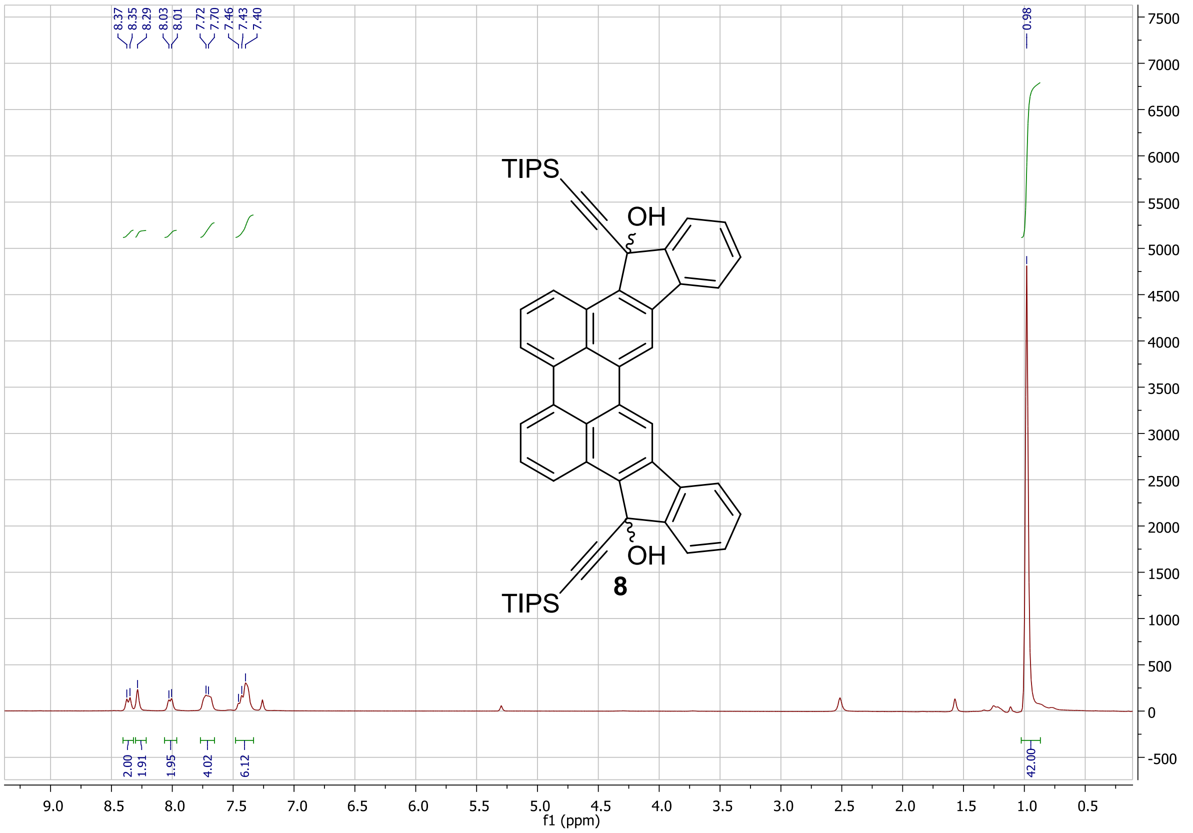

8.37
8.35
8.29
8.03
8.01
7.72
7.70
7.46
7.43
7.40
0.98
TIPS
OH
8
2.00
1.91
1.95
4.02
6.12
42.00
f1 (ppm)

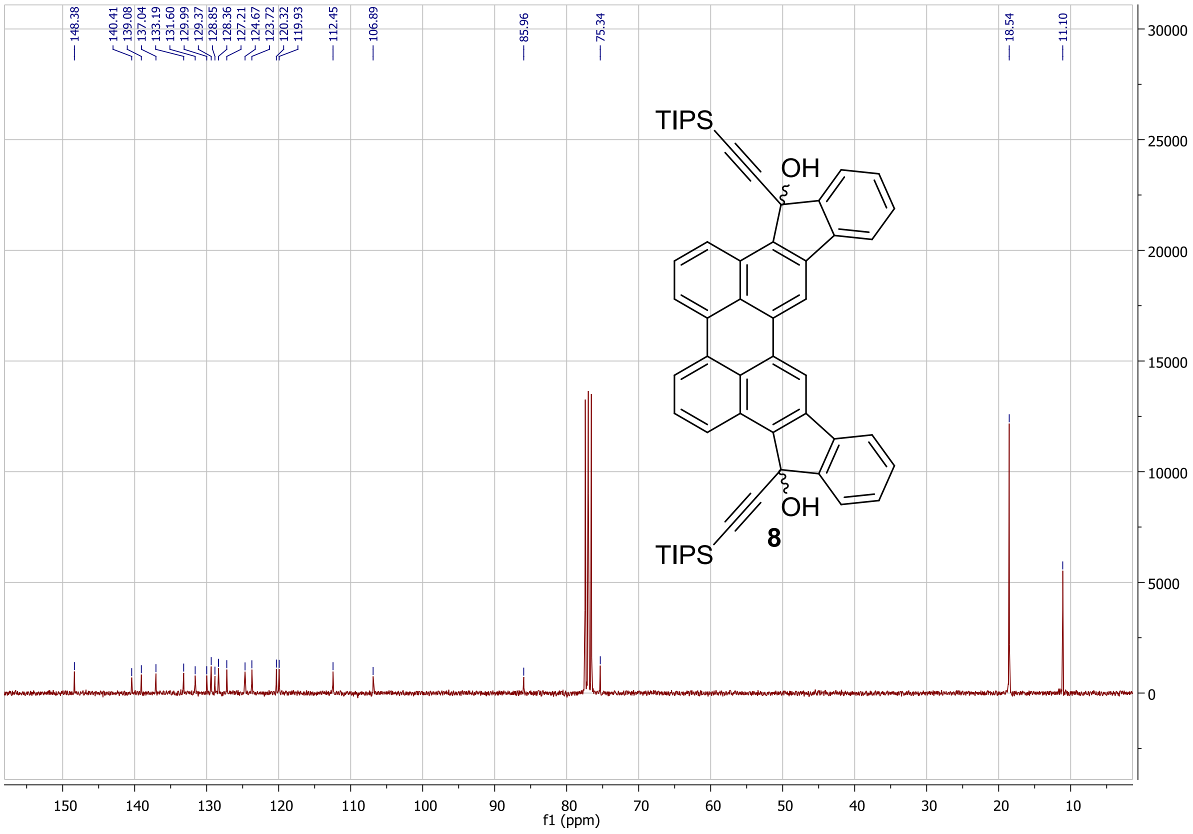

148.38
140.41
139.08
137.04
133.19
131.60
129.99
129.37
128.85
128.36
127.21
124.67
123.72
120.32
119.93
112.45
106.89
85.96
75.34
18.54
11.10
TIPS
OH
8
f1 (ppm)

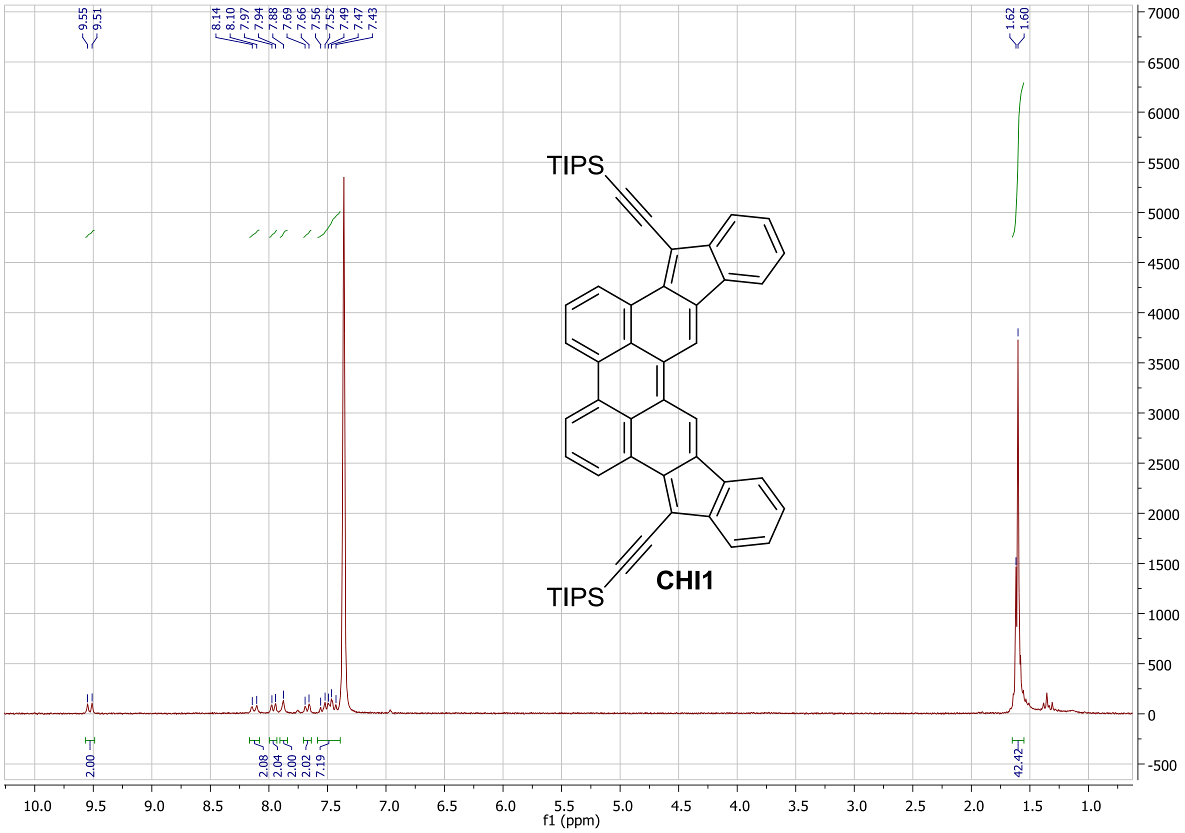
TIPS
TIPS
CHI1
f1 (ppm)

IR Spectra

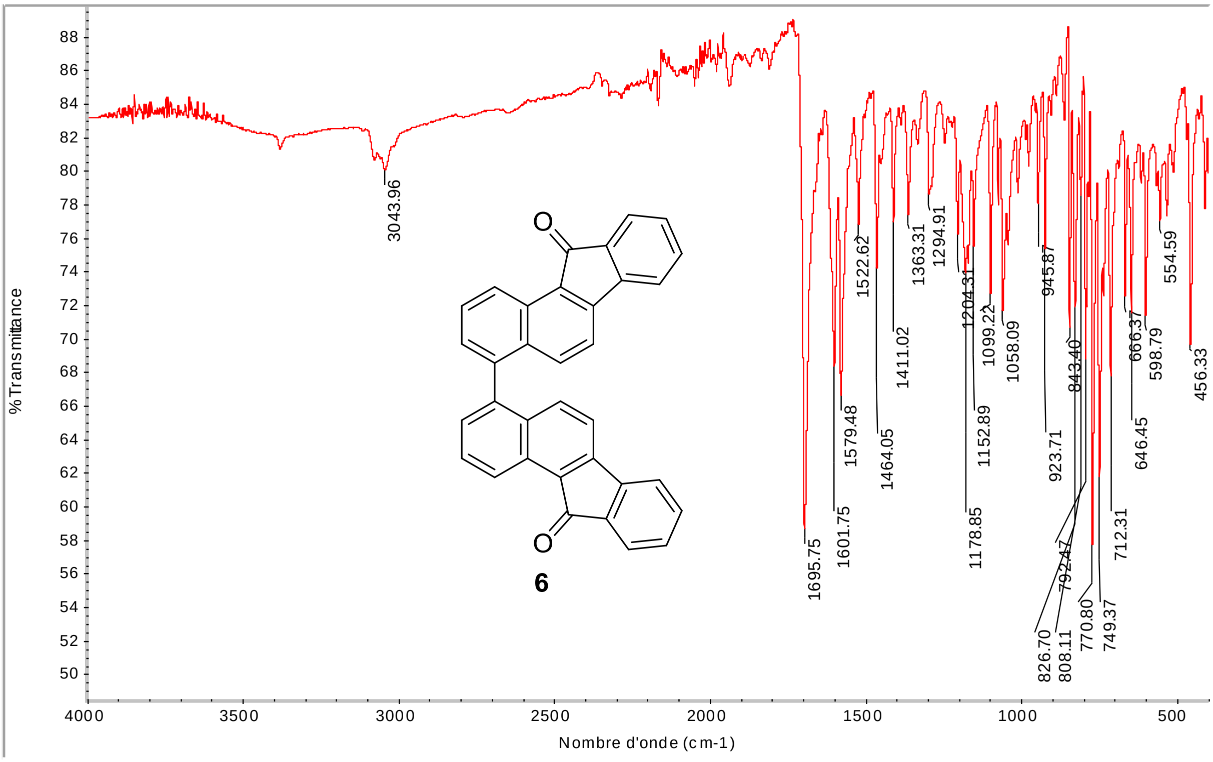
% Transmittance
Nombre d'onde (cm-1)
3043.96
1695.75
1601.75
1579.48
1522.62
1464.05
1411.02
1363.31
1294.91
1204.31
1178.85
1152.89
1099.22
1058.09
945.87
923.71
843.40
826.70
808.11
792.47
770.80
749.37
712.31
666.37
646.45
598.79
554.59
456.33
6

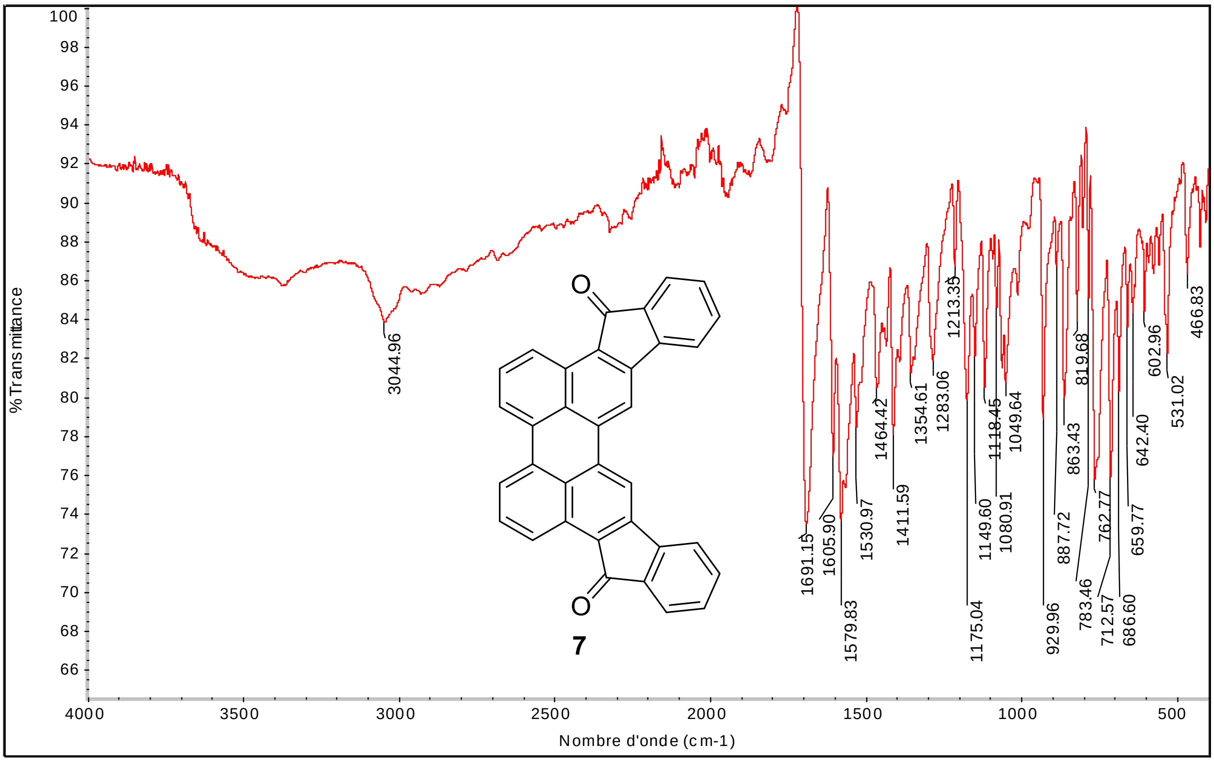
% Transmittance
Nombre d'onde (cm-1)
3044.96
1691.15
1605.90
1579.83
1530.97
1464.42
1411.59
1354.61
1283.06
1213.35
1175.04
1149.60
1118.45
1080.91
1049.64
929.96
887.72
863.43
819.68
783.46
762.77
712.57
686.60
659.77
642.40
602.96
531.02
466.83
O
O
7

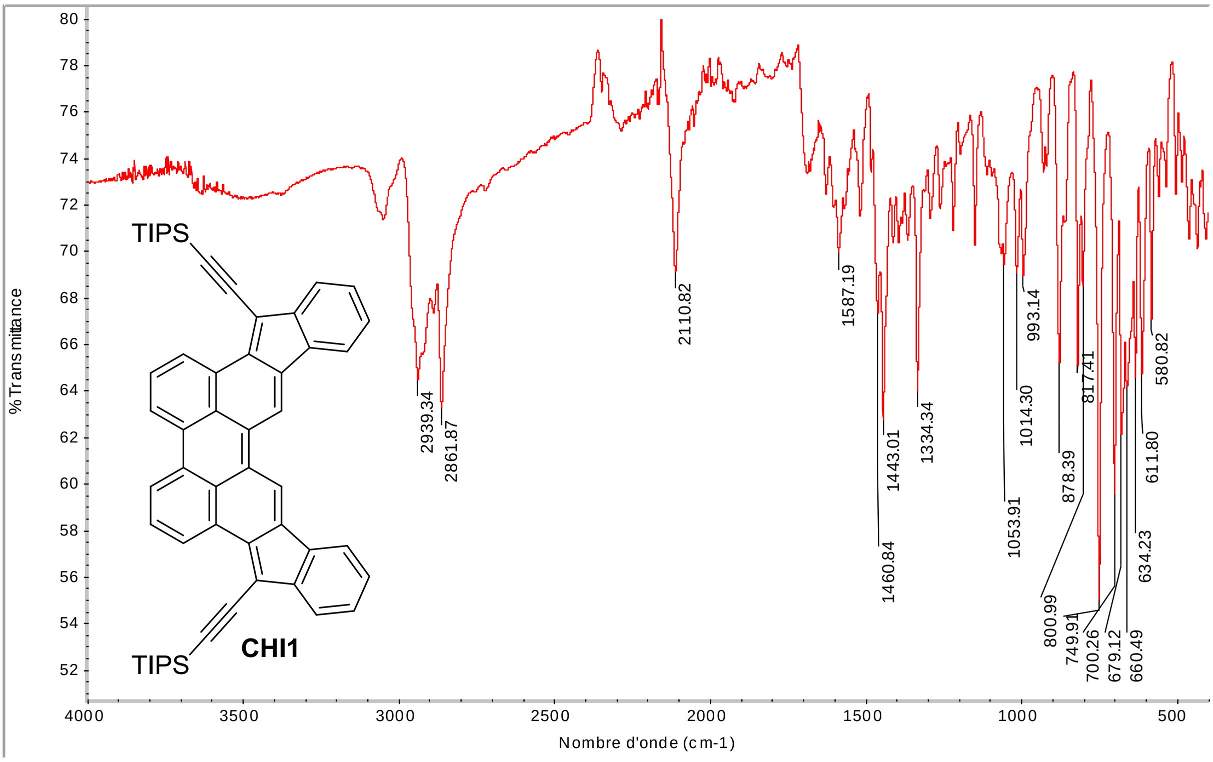
% Transmittance
Nombre d'onde (cm-1)
2939.34
2861.87
2110.82
1587.19
1460.84
1443.01
1334.34
1053.91
1014.30
993.14
878.39
817.41
800.99
749.91
700.26
679.12
660.49
634.23
611.80
580.82
TIPS
TIPS
CHI1

OFET Fabrication and Characterization

Solution-processed OFETs, bottom-gate/top-contact

Bottom-gate/top-contact (BG/TC) OFETs for were fabricated on heavily doped n-type silicon wafers covered with thermally grown silicon dioxide (200 nm) which was cleaned by piranha solution. The silicon dioxide acts as a gate dielectric layer, and the silicon wafer serves as a gate electrode. The HMDS (hexamethyldisilazane) treatment was carried out by immersing the substrate in HMDS at room temperature for >10 h. Solution processed organic semiconductor layers were formed by spin-coating at 2000 rpm 60 s from a 0.3 wt% chloroform solution, followed by thermal annealing at 60 °C for ca. 30 min. Gold was used as source and drain electrodes (50 nm) and deposited on the organic semiconductor layer through a shadow mask with L/W = 50/2000 μm.

Solution-processed OFETs, Tog-gate/bottom-contact

Tog-gate/bottom-contact (TG/BC) OFETs were constructed on glass substrates with the cross-linked PVP (poly-4-vinylphenol), which was prepared by spin-coating from a solution of PVP (Mw ~5000, 10.0 wt%) and poly(melamine-co-formaldehyde) (Mn ~432, 10.0 wt%) in propylene glycol monomethyl ether acetate (PGMEA) at the rotational speed of 500 rpm for 5 s and then 4000 rpm for 60 s, followed by the cross-linkage at temperatures of 150 °C for 60 min under nitrogen atmosphere. Bottom-contact gold source-drain electrodes (50 nm) were deposited on PVP through a shadow mask with L/W = 50/2000 μm. The self-assembled monolayer (SAM) treatment for source-drain electrodes was prepared by immersing the substrates in a 30 mM isopropyl alcohol solution of pentafluorobenzenethiol (PFBT) for 5 min at room temperature. The substrates were then rinsed with pure propanol and blown dry with nitrogen. A 1 wt% solution of fluoropolymer (DuPont™, Teflon® AF 1600,) in Fluorinert™ (3M™ FC-43) was used as a bank layer around source-drain electrodes and printed by using dispenser equipment (Musashi Engineering, Image Master 350 PC) that included a three-axis table and an air dispenser, both of which were computer-controlled to dispense and pattern the solution. 200-nm-thick fluoropolymer bank layers were printed at a patterning speed of 20 mm/s and discharge pressure of 7 kPa, and they were subsequently cured at 40 °C for 5 min in air. Organic semiconductor layers were formed by drop-casting from a 0.1–0.2 wt% solution of compounds **CHI1** with/without 0.025–0.05 wt% of polystyrene (PS) in mesitylene, followed by thermal annealing at 60 °C for ca. 1 h. The CYTOP dielectric layer (435–600 nm measured by DektakXT™ Stylus Profiler) was spin-coated from a solution of CYTOP™ CTL-809M in CT-solv 180 from Asahi Glass at the rotational speed of 500 rpm for 5 s and then 1800 rpm for 120 s on top of the organic layer and then was dried at room temperature for 2 h. The Al gate electrode (50 nm) was formed by vacuum evaporation through a shadow mask.

The OFET measurements were carried out at room temperature in a glovebox without exposure to air with a semiconductor parameter analyzer (4200-SCS, KEITHLEY). Mobilities (μ) were calculated in the saturation regime by the relationship: $\mu_{sat} = (2I_D L)/[WC_i(V_G - V_{th})^2]$ where I_D is the source-drain saturation current; C_i is the capacitance of the insulating layer; V_G is the gate voltage and V_{th} is the threshold voltage. The latter can be estimated as the intercept of the linear section of the plot of V_G $(I_D)^{1/2}$**.**

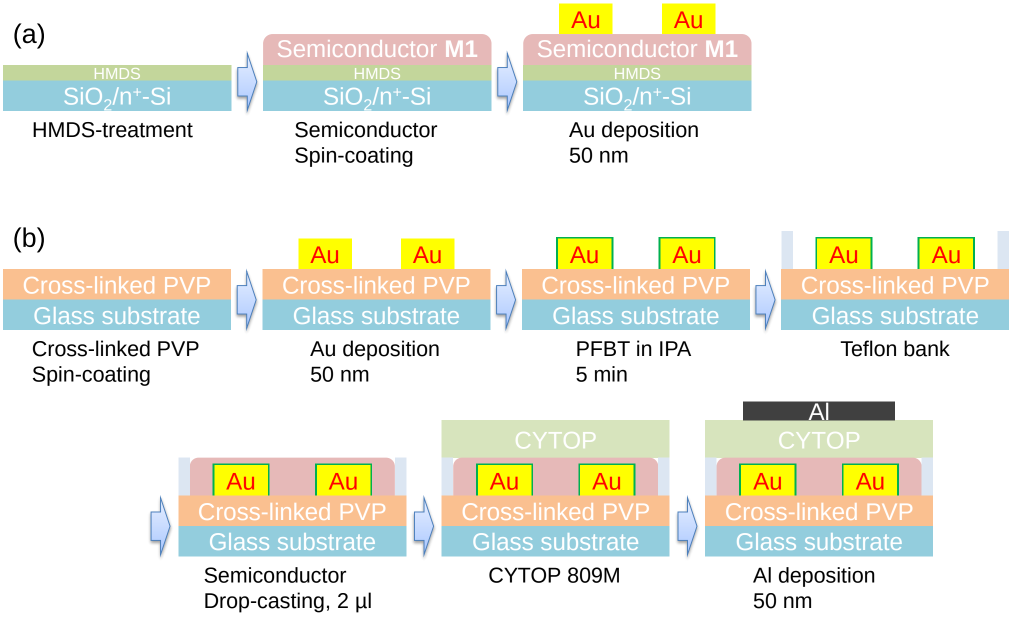

Figure S3 Schematic illustrations of the fabrication processes for (a) bottom-gate/top-contact (BG/TC) OFETs and (b) top-gate/bottom-contact (TG/BC) OFETs.

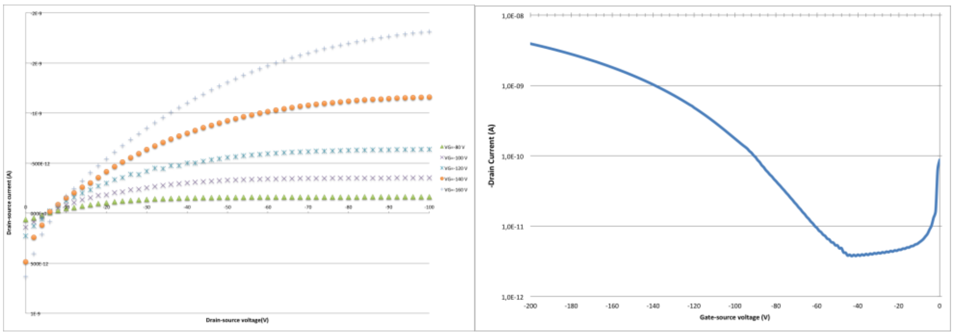

Figure S4 OFET and transfer characteristics of a device made with **7** as active layer on PMMA/ITO/glass substrate.

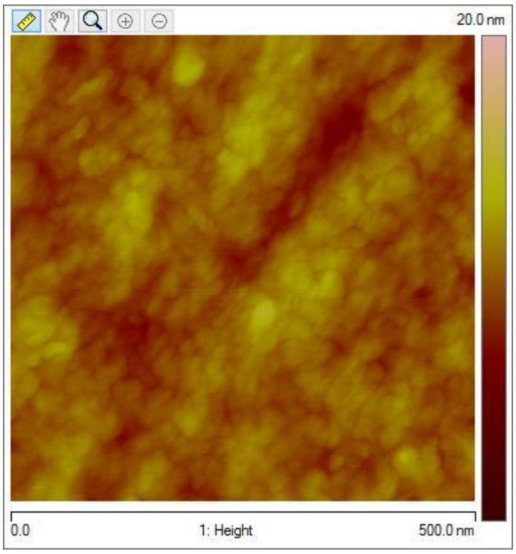

Figure S5 Atomic force microscopy height image (0.5 μm X 0.5 μm) of the diindeno[1,2-*b*:2',1'-*n*]perylene-5,12-dione (**7**) film deposited on Si-wafer.

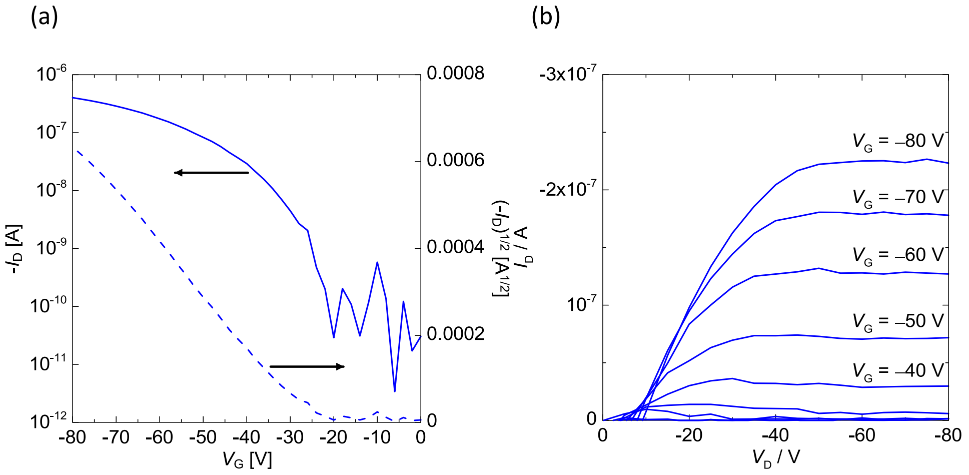

Figure S6 (a) Transfer and (b) output characteristics of BG/TC device for thin film of **CHI1**.

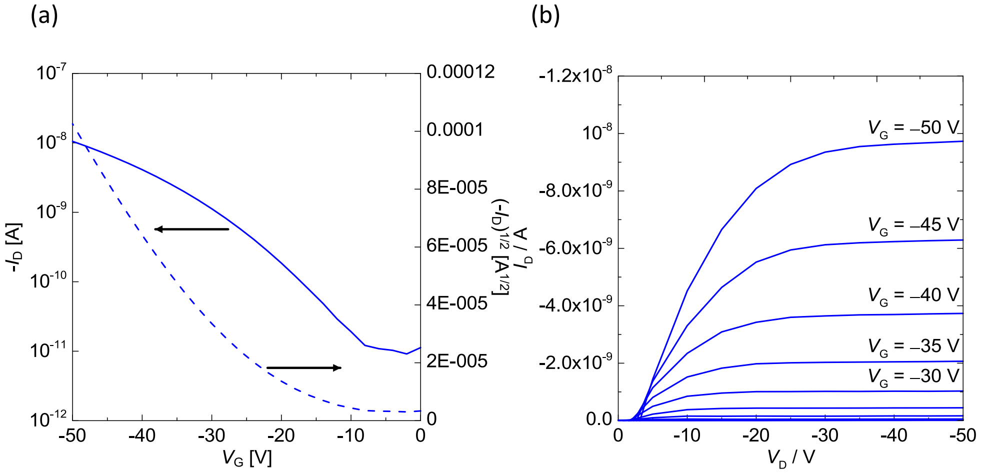

Figure S7 (a) Transfer and (b) output characteristics of TG/BC device for thin film of **CHI1** deposited by drop-casting from the 0.2 wt% solution in the mesitylene.

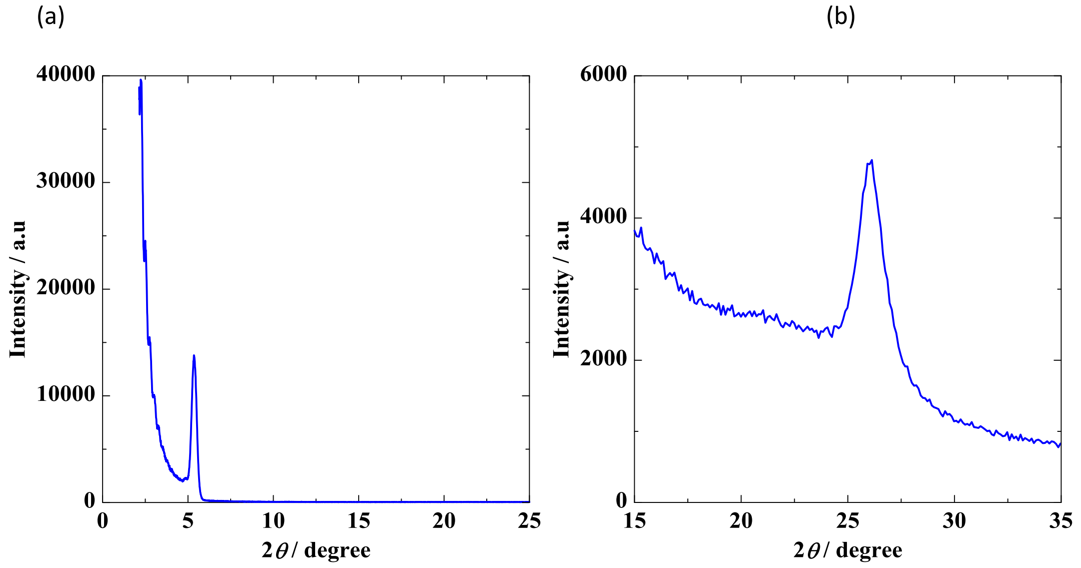

Figure S8. (a) Out-of-plane and (b) in-plane X-ray diffractograms of thin film of **CHI1** spin-coated from the chloroform solution and deposited on HMDS-treated SiO_2 substrate.
